# Association between circulating inflammatory biomarkers and functional outcome or perihaematomal oedema after ICH: a systematic review & meta-analysis

**DOI:** 10.12688/wellcomeopenres.19187.2

**Published:** 2023-11-21

**Authors:** Caoimhe Kirby, Jack Barrington, Lotte Sondag, James J.M. Loan, Floris H.B.M. Schreuder, Barry W. McColl, Catharina J.M. Klijn, Rustam Al-Shahi Salman, Neshika Samarasekera

**Affiliations:** 1 Centre for Clinical Brain Sciences, The University of Edinburgh, Edinburgh, Scotland, UK; 2 Centre for Discovery Brain Sciences, The University of Edinburgh, Edinburgh, Scotland, UK; 3 UK Dementia Research Institute, The University of Edinburgh, Edinburgh, Scotland, UK; 4 Department of Neurology, Donders Institute for Brain, Cognition and Behaviour, Radboud Universiteit, Nijmegen, Gelderland, The Netherlands

**Keywords:** Intracerebral Haemorrhage, Inflammation, Perihematomal Oedema, Biomarkers, Neuroimmunology, Stroke

## Abstract

**Background:**

Currently, there are no specific medical treatments for intracerebral haemorrhage (ICH), but the inflammatory response may provide a potential route to treatment. Given the known effects of acute brain injury on peripheral immunity, we hypothesised that inflammatory biomarkers in peripheral blood may be associated with clinical outcome following ICH, as well as perihaematomal oedema (PHO), which is an imaging marker of the neuroinflammatory response.

**Methods:**

We searched OVID Medline and EMBASE on 07 April 2021 for studies of humans with ICH measuring an inflammatory biomarker in peripheral blood and PHO or clinical outcome. Risk of bias was assessed both by using a scale comprising features of the Newcastle-Ottawa Assessment Scale, STROBE-ME and REMARK guidelines, and for studies included in meta-analysis, also by the QUIPS tool.We used random effects meta-analysis to pool standardised mean differences (SMD) if ≥1 study quantified the association between identical biomarkers and measures of PHO or functional outcome.

**Results:**

Of 8,615 publications, 16 examined associations between 21 inflammatory biomarkers and PHO (n=1,299 participants), and 93 studies examined associations between ≥1 biomarker and clinical outcome (n=17,702 participants). Overall, 20 studies of nine biomarkers (n=3,199) met criteria for meta-analysis of associations between inflammatory biomarkers and clinical outcome. Death or dependency (modified Rankin Scale (mRS) 3‒6) 90 days after ICH was associated with higher levels of fibrinogen (SMD 0.32; 95%CI [0.04, 0.61]; p=0.025), and high mobility group box protein 1 (HMGB1) (SMD 1.67; 95%CI [0.05, 3.30]; p=0.04). Higher WBC was associated with death or dependency at 90 days (pooled SMD 0.27; 95% CI [0.11, 0.44]; p=0.001; but the association was no longer significant when the analysis was restricted to studies with a low risk of bias (pooled SMD 0.22; 95% CI -0.04-0.48). Higher CRP seemed to be associated with death or dependency at 90 days (pooled SMD 0.80; 95% CI [0.44, 1.17]; p<0.0001) but this association was no longer significant when adjusted OR were pooled (OR 0.99 (95% CI 0.98-1.01)).

**Conclusions:**

Higher circulating levels of, fibrinogen and HMGB1 are associated with poorer outcomes after ICH. This study highlights the clinical importance of the inflammatory response to ICH and identifies additional research needs in determining if these associations are mediated via PHO and are potential therapeutic targets.

**Registration:**

PROSPERO ( CRD42019132628; 28/05/2019).

## Introduction

Spontaneous intracerebral haemorrhage (ICH) accounts for 16–30% of all stroke cases ^1^. Overall, 40% of patients die within one month after ICH with only 12–39% living independently after six months ^2^. There are currently no specific medical treatments that have proven benefit to improve outcome ^3^.

There is growing interest in the inflammatory reaction after ICH since it may be a therapeutic target ^4^. Perihaematomal oedema (PHO) is thought to be an imaging biomarker of the neuroinflammatory response ^5^ and neuroinflammation may lead to PHO development. PHO is thought evolve in stages with initial cytotoxic oedema leading to vasogenic oedema ^5^. Inflammatory biomarkers are highly expressed in human brain tissue following ICH ^6^ and several ongoing clinical trials are targeting the immune system ^7^. In animal models, local and recruited immune cells release inflammatory mediators ( *e.g.*, cytokines, matrix-metalloproteinases (MMPs), and damage associated molecular patterns (DAMPs)) that contribute to brain damage and repair ^8^. Various immunomodulatory treatments have been shown to improve outcome in preclinical models of ICH ^9^. In humans, stroke-induced changes to the peripheral immune system are associated with the development of sequelae. Circulating immune cell characteristics are associated with trajectory of post-stroke cognitive impairment ^10^ and fewer circulating leukocytes are associated with infection following ICH ^11^. Since PHO is an imaging marker of neuroinflammatory response, we hypothesised that circulating inflammatory biomarkers associated with clinical outcome should also be associated with measures of PHO.

We therefore conducted a systematic review and meta-analysis of associations between circulating inflammatory biomarkers and either PHO or functional outcome after ICH. We believe this is the first systematic review and meta-analysis that has assessed the relationship between inflammatory biomarkers and PHO and/or functional outcome after ICH without restriction based on pre-specified biomarkers. We aimed to (i) identify all studies examining associations between one or more blood-based biomarker and PHO or functional outcome after ICH, (ii) quantify pooled associations between individual biomarkers and PHO or outcome where possible, and (iii) determine whether study-level variables modified any of these associations.

## Methods

We performed a systematic review and meta-analysis. The study protocol was pre-registered with the International prospective register of systematic reviews (PROSPERO; CRD42019132628; 28 May 2019). This systematic review is reported in line with the PRISMA guidelines ^12^.

### Search strategy and study selection

**Figure 1.**
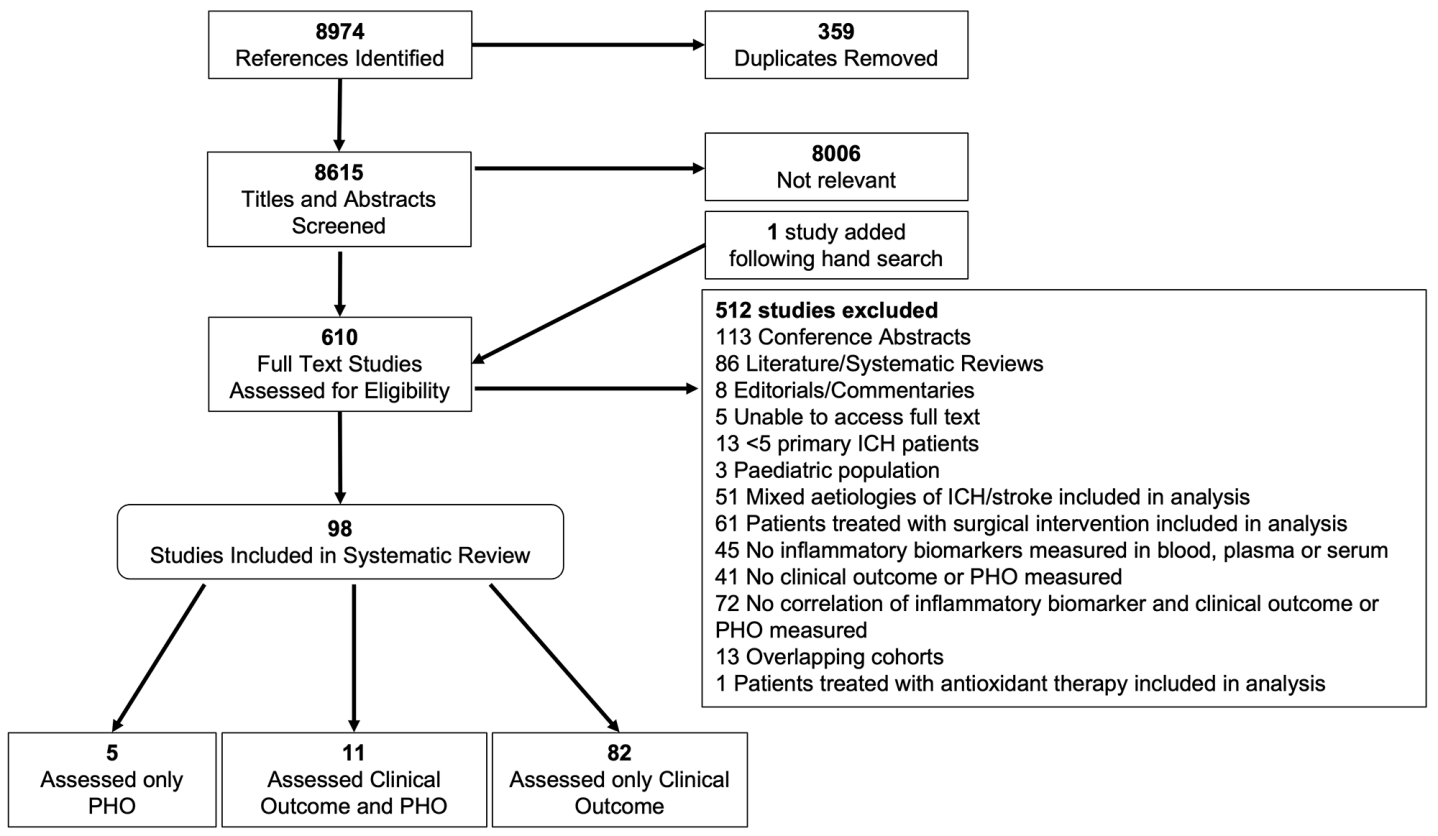
Flow diagram of study selection for inclusion in systematic review and meta-analysis including the rationale for exclusion. PHO, perihaematomal oedema; ICH, intracerebral haemorrhage.

CK searched Ovid MEDLINE (1946) (RRID:SCR_002185) and Ovid EMBASE (1974) (RRID:SCR_001650) on 07 April 2021 using a prespecified search strategy that consisted of terms to identify studies that measured inflammatory markers in the blood of ICH patients and assessed functional outcome and/or PHO (Supplemental Information 1, available as *Extended data* ^13^). The search was not limited by language or publication date. After automated de-duplication in Covidence (covidence.org), three authors (CK, LS, JB) independently screened titles and abstracts to identify potentially eligible studies and read the full text of articles that were potentially eligible for inclusion. Corresponding authors were contacted if full-text articles could not be obtained. A third independent reviewer (FHBMS, JL or NS) made the final decision over inclusion when conflicts arose at the abstract or full-text screening stage ( Figure 1).

### Eligibility criteria

We included observational studies of ≥5 adults (≥16 years of age) with spontaneous ICH where inflammatory markers were measured in the blood, serum or plasma. We selected biomarkers if they were a marker ( *e.g.*, C-reactive protein) or a mediator ( *e.g.*, cytokines) of the inflammatory response and grouped the inflammatory biomarkers into six categories based on broad biological activity ^14^. Categories were defined based on discussions between domain experts (JB, BM): immune cells, acute phase reactants (defined as liver-produced plasma proteins ^15^), cytokines/chemokines, damage-associated molecular patterns (DAMPs-defined here as molecules released from intracellular compartments during inflammation ^16^), tissue remodelling factors and adhesion molecules. We excluded: 1) studies of ICH due to an underlying macrovascular cause, traumatic ICH, ICH due to hereditary cerebral amyloid angiopathy or studies of ICH with mixed causes, 2) stroke cohorts where spontaneous ICH cases could not be separated from non-ICH cases, 3) studies including surgically treated patients that could not be separated from non-surgically treated cases, 4) conference abstracts, systematic or narrative reviews. Where studies had overlapping cohorts, the study with the largest cohort was included.

### Data extraction

A minimum dataset (the biomarker, outcome definition, time point of biomarker and outcome measurement, measure of association between biomarker and outcome and risk of bias) was extracted from all included studies by one reviewer (CK, JB or NS) and is summarized in a narrative synthesis (Supplemental Table 2, *Extended data* ^13^). Two authors (CK, JB, or NS) used a standardized proforma to independently extract data from all studies included in meta-analysis (Supplemental Information 2 ^13^). Conflicts were arbitrated by a third reviewer (JB, NS). As the included studies were mostly case series with no control group, risk of bias was assessed by one reviewer (CK, JB, NS) using an eight-item composite scale comprising features of the Newcastle-Ottawa Assessment Scale ^17^ (case definition, representativeness of the cases), STROBE-ME ^18^ and REMARK ^19^ guidelines. The assessment assigned points for features indicating a high risk of bias, ranging from 0 (no risk of bias) to 8 (high risk of bias) (Supplemental Information 3 in *Extended data* ^13^). Conflicts relating to risk of bias were assessed by the same arbitrator that arbitrated conflicts relating to data extraction (JL, FS). The risk of bias of studies included in one or more meta-analysis were additionally assessed using the QUIPS tool ^20^ (Supplemental Table 1 in Extended Data).

### Meta-analysis

We undertook a meta-analysis of associations between a biomarker and outcome if (i) the same biomarker was assessed in ≥2 studies, (ii) means/medians (and errors) of biomarkers were reported and (iii) the same outcome measure was reported between studies at the same time point after ICH onset. We used the *metafor* (RRID:SCR_003450) (version 3.0-2) and *tidyverse* (RRID:SCR_019186) packages in R Project for Statistical Computing (RRID:SCR_001905) (version 3.6.3) ^21^. Median and range were first converted to mean and standard deviation based on previously published methods ^22^. We then calculated individual standardized mean differences (SMDs) using Hedges g, before estimating inter-study variability (τ ^2^) using a restricted maximum likelihood random-effects model, generating a summary standardized mean difference. We used SMD since this was the most commonly reported outcome measure. However we also used the stata command metan to perform meta-analysis of odds ratios where they were provided. We assessed heterogeneity using the Higgins’ I ^2^ statistic and Q statistics. Where more than three studies were included in meta-analysis, we evaluated outliers both graphically ^23^ and *via* influence diagnostics ^24^ and if we identified an outlier, sensitivity was assessed by leave-one-out analysis. Publication bias was evaluated graphically with funnel plots and Egger’s regression test ^25^ where more than 10 studies were included in meta-analysis. If more than 10 studies were included in a meta-analysis, we used meta-regression to evaluate the influence of the pre-defined moderators ICH volume and age on the observed model variances. If more than 10 studies were included in meta-analysis, studies were stratified into high-quality (0–1 risk of bias score) and low quality (≥2 risk of bias score) according to the eight item composite scale and sub-group analysis performed to determine the impact of study quality on summary estimates. We have also stratified studies into low, moderate or high risk of bias according to the QUIPS rating tool and once again performed subgroup analyses to determine the impact of study quality on summary estimates. I ^2^ values of 0–39% were considered small, 40–69% moderate and 70–100% high. The code to reproduce the meta-analysis can be found at *Extended data* ^26^.

## Results

Our search yielded 8,794 articles, of which 8,615 were unique. In total, 98 studies of 50 unique inflammatory biomarkers in 18,000 participants were included in our narrative synthesis, of which 93 (n=17,702 participants) examined the association between at least one biomarker and clinical outcome. Overall, 11 of these also examined PHO (n=1,001 participants). Five studies (n=298) examined an association between biomarkers and PHO alone ( Figure 1).

From all 98 included studies, 85 (87%) studies reported biomarker levels on admission or within 24 hours from ICH onset, of which 13 (15%) also reported biomarkers at later time points. A total of 12 (12%) studies only reported biomarker levels at later time points ranging from <48 hours to 30 days after ICH onset, and one study did not report biomarker time point. Overall, 37 (38%) studies reported on a single biomarker, and the remainder reported on two biomarkers (n=21, 21%), three biomarkers (n=14, 14%) or four or more biomarkers (n=26, 27%).

### Narrative synthesis of the associations between inflammatory biomarkers and perihaematomal oedema

In total, 16 studies ^27– 42^ of 1,299 participants assessed the relationship between 21 inflammatory biomarkers and PHO (14 by CT, two by MRI) ( Table 1; participant characteristics listed in Supplemental Table 3 ^13^), of which we excluded one study ^31^ which assessed PHO using midline shift only. The median risk of bias was 2 [0–3]. Due to broad heterogeneity in the method and timing of PHO measurements between studies coupled with variation in the ways in which studies assessed the relationship between a given biomarker and PHO, we could not perform a meta-analysis of the association between inflammatory mediators and PHO. Therefore, what follows is a narrative synthesis of all published articles that analysed the association between one or more inflammatory biomarkers and PHO ( Table 1).

**Table 1.** Associations between circulating inflammatory biomarkers and perihaematomal oedema. PHO, perihaematomal oedema; TIMP, Tissue Inhibitors of Metalloproteinases; CRP, C-reactive protein; ICAM-1, Intracellular Adhesion Molecule 1; VCAM-1, Vascular Cell Adhesion Molecule 1; S100B, S100 Calcium Binding Protein B; NLR, neutrophil-to-lymphocyte ratio; WBC, white blood cell; MRI, Magnetic Resonance Imaging; ICH, intracerebral haemorrhage; EVD, External Ventricular Drain; GCS, Glasgow Coma Scale.

Biomarker	Study	Sample size	Measurement of PHO volume	Relationship between biomarker and PHO volume
**MMP-9**				
MMP-9	Abilleira (2003)	38 ^#a^	Absolute and relative	positive correlation in patients with deep ICH but lost in multivariate analysis ^*1^; no association with lobar ICH
MMP-9	Alvarez-Sabin (2004)	21	Absolute, PHO enlargement	positive correlation with baseline absolute PHO volume, positive correlation with PHO enlargement within the first 48 h
MMP-9	Castellazzi (2010)	28	absolute	positive correlation at 24h and 48h
MMP-9	Howe (2018)	79	Not specified	no association **
**Other MMPs**				
MMP-2	Castellazzi (2010)	28	absolute	positive correlation at 24h and negative correlation at 7 days
MMP-3	Howe (2018)	79	Not specified	no association **
MMP-8	Howe (2018)	79	Not specified	positive correlation at day 6-8
**TIMPs**				
TIMP-1	Abilleira (2003)	38 ^#a^	absolute and relative	no association **
TIMP-1	Alvarez-Sabin (2004)	21	absolute, PHO enlargement	negative correlation with baseline absolute PHO volume and PHO enlargement over 7 days
TIMP-1	Castellazzi (2010)	28	absolute	no association **
TIMP-2	Castellazzi (2010)	28	absolute	no association **
MMP-9/ TIMP-1 ratio	Alvarez-Sabin (2004)	21	absolute, PHO enlargement	associated with absolute PHO 12h after symptom onset
**Ferritin**				
Ferritin	Bakhshayesh (2014)	63	PHO growth	positive correlation with PHO growth over 72hrs ^*2^
Ferritin	Mehdiratta (2007)	23	relative	positive correlation on day 3-4 but not at baseline
Ferritin	Perez de la Ossa (2010)	92	absolute and PHO growth	positive correlation with PHO volume at baseline, 24h and 7 Days. no correlation with PHO growth at 72h or day 7
**Interleukins**				
IL-4	Castillo (2002)	116 ^#b^	absolute	no association at day 3-4 or 3 months
IL-4	Wang (2016)	94	absolute	negative correlation
IL-6	Castillo (2002)	116 ^#b^	absolute	positive association at day 3-4 and 3 months but lost in multivariate analysis ^*3^
IL-6	Wang (2016)	94	absolute	positive correlation
IL-8	Wang (2016)	94	absolute	negative correlation
IL-10	Castillo (2002)	116 ^#b^	absolute	no association at days 3-4 ^*3^ or 3 months
IL-10	Wang (2016)	94	absolute	positive correlation
**TNFα, ICAM-1, VCAM-1, s100B**				
TNFα	Castillo (2002)	116 ^#b^	absolute	positive association at day 3-4 which remains in multivariate analysis, positive association at 3 months but not in multivariate analysis ^*3^
ICAM-1	Castillo (2002)	116 ^#b^	absolute	positive association at day 3-4 and 3 months but lost in multivariate analysis ^*3^
VCAM-1	Castillo (2002)	116 ^#b^	absolute	no association
S100B	Delgado (2006)	78	absolute	positive correlation at day 3
**White blood cell count, Lymphocyte count, Neutrophil count, NLR**				
NLR	Chen (2020)	6	NS	positive correlation at day 7
NLR	Gusdon (2017)	153	PHO growth	positive correlation at 24h ^*4^
NLR	Volbers (2018)	292	peak PHO on any CT scan	positive correlation with peak PHO volume at day 6 in univariate analysis
Lymphocyte count	Volbers (2018)	292	peak PHO on any CT scan	reduced lymphocyte count on day 4 is an independent predictor in multivariate analysis ^*5^
WBC	Mehdiratta (2007)	23	relative	no association
WBC	Gusdon (2017)	153	PHO growth	no association ^*4^
**MRI Studies**				
MMP-9	Li (2013)	59	absolute	no association
MMP-3	Li (2013)	59	absolute	positive association ^*6^
VEGF	Li (2013)	59	absolute	no association
WBC	Venkata- subramanian (2011)	22 ^#c^	relative and peak relative PHO	no change in median WBC count in patients with higher rPHO or peak rPHO

# ^a^ PHO measured in 38 (total cohort n=57) # ^b^ Biomarkers measured in 116 (total cohort n=124) # ^c^ PHO measured in 22 (total cohort n=27) *Variables included in multivariate analysis: * ^1^: Abilleira (2003) : ICH volume, MMP-9, Canadian stroke scale score, smoking, chronic alcoholism * ^2^: Bakhshayesh (2014): variables not stated * ^3^: Castillo (2002): admission Canadian stroke scale score, temperature, ICAM-1, IL-6, IL-10, glutamate * ^4^ Gusdon (2017): ICH Volume, intraventricular extension, EVD use, GCS * ^5^ Volbers (2018): ICH volume, age, haematoma location * ^6^ Li (2013): ICH volume, age, gender ** narrative only

The most frequently measured biomarker was MMP-9, which was assessed in five studies (CT n=4 ^34– 37^, MRI n=1 ^28^; n=225), three ^35– 37^ of which found that MMP-9 levels were positively associated with PHO volume in univariate analyses only. MMP-2 ^37^ and MMP-8 ^34^ were positively associated with absolute PHO volume. Comparisons of MMP-3 and PHO were inconsistent with one study finding no association ^34^ and one study showing a positive association ^28^. TIMP-1 was not associated with PHO in two studies ^35, 37^, but had an inverse association with PHO volume in one ^36^.

Higher levels of ferritin were associated with PHO growth ^32^, relative ^40^, or absolute PHO volume ^29^. White blood cell (WBC) count had no association with relative PHO volume ^39, 40, 42^, whereas neutrophil count and neutrophil-to-lymphocyte ratio (NLR) had a positive correlation with midline shift after adjusting for ICH volume and infection ^31^. NLR was positively correlated with PHO in three studies ^30, 38, 39^.

IL-4 was positively associated with absolute PHO in one of two studies ^41^. IL-6 was associated with absolute PHO volume in two studies ^33, 41^ and TNFα was associated with absolute volume in univariate but not multivariate analyses ^33^.

Adhesion molecules ICAM1 and VCAM1 were not associated with absolute PHO volume in multivariate analyses ^33^.

### Meta-analysis of the association between inflammatory biomarkers and clinical outcome

From 93 studies examining the association between inflammatory biomarkers and clinical outcome after ICH, the median risk of bias was two [1–3]. The most commonly reported outcome measure was the modified Rankin scale (mRS) (n=59, 63%) and the most frequent time point for outcome ascertainment was 90 days (n=41, 44%) (Supplemental Figure 1A and 1B ^13^). Therefore, studies reporting mRS at 90 days were selected for meta-analysis with poor outcome defined as death or dependency (mRS 3–6). Overall, 20 studies of nine biomarkers were included in meta-analysis ( Table 2). Of these 20 studies, only one study reported steroid use at the time of ICH ^43^ and no studies reported on the use of osmotic agents, NSAIDs, or other immunomodulatory drugs at the time of ICH. No studies reported the number of febrile patients on admission, but 13 reported the presence of intercurrent infection with 10 using it as an exclusion criterion. Seven studies reported the use of antiplatelet agents and nine studies reported anticoagulant medication at the time of ICH (Supplemental Table 4, available as *Extended data* ^13^). All 93 studies examining the association between inflammatory biomarkers and clinical outcome have been included in a narrative synthesis (Supplemental Table 2, available as *Extended data* ^13^).

**Table 2.** Information on the 20 studies that were included in a meta-analysis of inflammatory biomarkers with 90-day mRS. mRS, modified Rankin Scale; ICH, intracerebral haemorrhage; CRP, C-reactive protein; S100B, S100 Calcium Binding Protein B; WBC, white blood cell; HMGB1, high mobility group box protein 1; GCS, Glasgow Coma Scale.

Study	Biomarkers	Country	n	Age	Sex male, n (%)	ICH Location n(%) Lobar/Deep/ Infratentorial	ICH volume mL or cm3	Intraventricular haemorrhage n(%)	Admission GCS
Bakhshayesh (2014)	Ferritin	Iran	63	70 (12)	34 (54)	13(21)/43(68)/7(11)	29 (22)	29 (46)	13 [9-14]
Brea (2009)	S100B	Spain	44	62 (13)	NR (59)	NR/NR/0(0)	19 [10-30]	NR	NR
Castellanos (2005)	CRP, Fibrinogen, Neutrophil Count	Spain	138	70 (11)	NR (54)	38(28)/100(73)/0(0)	72 (48)	51 (37)	NR
Chen (2019)	CRP, WBC	China	106	67 [58–77]	NR (58)	26(25)/NR/ 4(13)	14 [7-21]	30 (28)	14 [13-15]
Delgado (2006)	S100B, WBC	Spain	78	75 [63–80]	53 (68)	20(26)/58(74)/0(0)	17 [4-38]	20 (25)	15 [14-15]
Fonseca (2021)	CRP	Portugal	135	73 [64–80]	69 (66)	58(43)/50(37)/27(20)	10 [2-26]	NR	14 [9-15]
He (2018)	CRP, Fibrinogen, Neutrophil Count, WBC	China	251	67 (8)	NR (56)	79(32)/131(53)/41(16)	10 [4-20]	103 (49)	12 [10-14]
Huangfu (2020)	CRP, Fibrinogen, WBC	China	159	64 [56–73]	NR (55)	0(0)/159(100)/0(0)	21 [14-28]	52 (33)	NR
Jiang (2014)	CRP, Fibrinogen, WBC	China	172	68 (10)	99 (58)	0(0)/172(100)/0(0)	32 (14)	61 (36)	NR
Lei (2020)	CRP, HMGB1, Neutrophil Count, WBC	China	240	56 (12)	131(55)	NR/NR/NR	26 (9)	68 (28)	198 (83%) at 15-13, 42 (17%) at ≤12
Mrackova (2020)	IL-6, S100B	Czech Republic	70	69 (12)	47(67)	NR/ NR/NR	16 [39]	NR	NR
Perez de la Ossa (2009)	Ferritin, Fibrinogen, IL-6, TNFα, WBC	Spain	92	68 (10)	61(66)	38(41)/54(59)/0(0)	20(12)	26 (28)	14 (1)
Rodriguez-Castro (2019)	CRP, Fibrinogen, WBC	Spain	961	74 (13)	549 (57)	364(38)/494(51) 85(9)	42 (36)	18 (2)	NR
Sagar (2021)	CRP	India	250	55 (13)	162 (65)	234(94)/16(6)/ 20(8)	32 (20)	135 (54)	8 (4)
Tang (2014)	WBC	Taiwan	43	60 (15)	28 (65)	NR/NR/NR	NR	NR	NR
Wang (2020)	CRP, WBC	China	106	67 (10)	60 (57)	26(25)/65(61)/15(14)	14 [6-22]	27 (26)	NR
Xiong (2015)	IL-6, TNFα, WBC	China	81	61 (11)	48 (59)	NR/54(67)/NR	20 (12)	18 (22)	14 (1)
Zhang (2020)	CRP, WBC	China	104	67 [57–76]	57 (55)	25(24)/NR/14(14)	12 [6-22]	26 (25)	15 [12 - 15]
Zhou (2010)	Fibrinogen, HMGB1, IL-6, TNFa, WBC	China	60	66 (9)	37 (62)	26(44)/NR/NR	24(13)	19 (32)	13 (2)
Zhou (2016)	S100B	China	46	68 (12)	30 (65)	8(17)/38(83)/0(0)	29 (13)	NR	NR

Data presented as mean (SD) or media [IQR] NR = Not Reported

**Figure 2.**
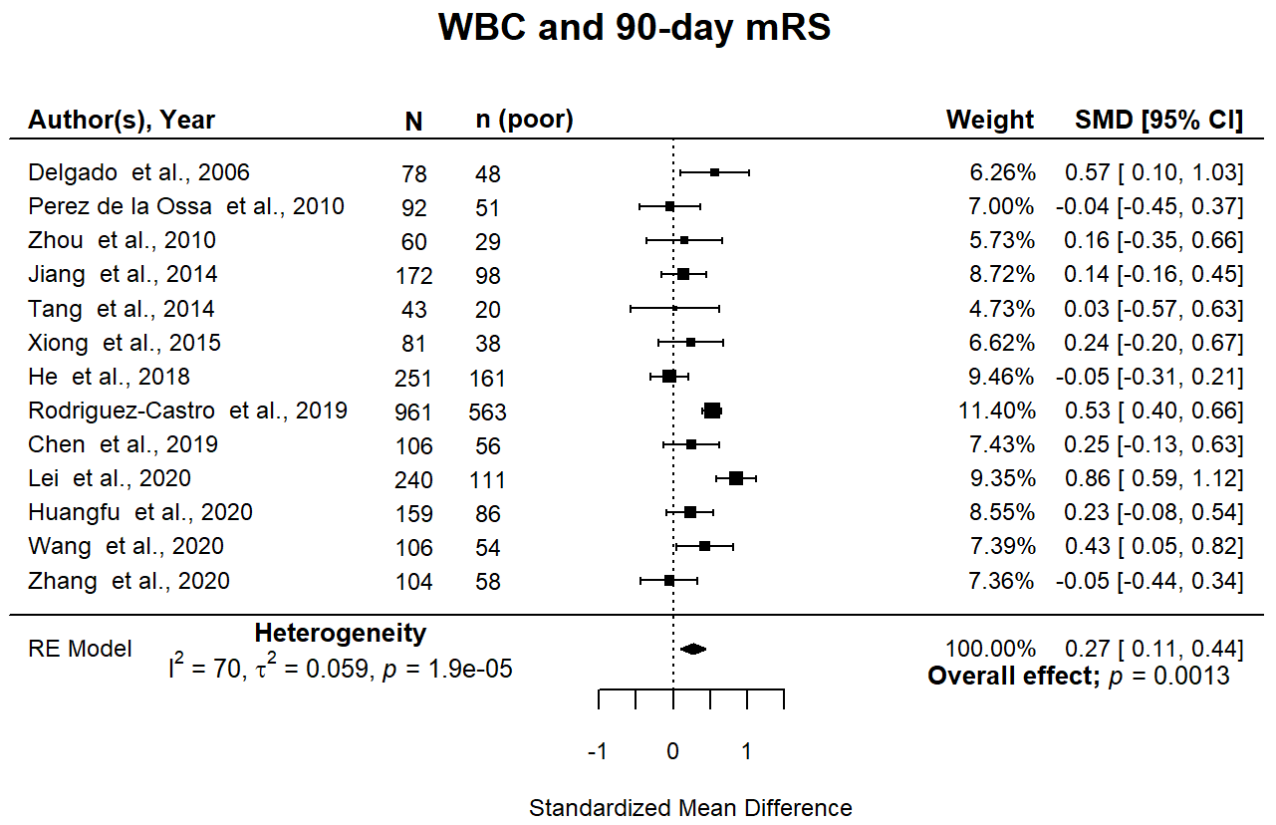
Forest plot of pooled associations of WBC with death or dependency 90 days after ICH. Pooled association of circulating WBC with death or dependency at 90 days. Poor outcome defined as mRS 3–6. WBC, white blood cell; ICH, intracerebral haemorrhage; mRS, modified Rankin Scale; SMD, standardised mean difference.

***Immune cells.*** A total of 53 studies reported WBC count ^27– 29, 44– 93^ in relation to clinical outcome after ICH. Of these, 13 ^27, 29, 49, 55, 58, 59, 64, 78, 82, 84, 86, 91, 93^ studies of 2,453 participants were meta-analysed. Overall, 10 (77%) studies reported WBC at admission, the remainder reporting it within 48 hours after ICH onset. One study ^64^ (7.7%) included a multivariate analysis. Higher WBC was associated with death or dependency at 90 days (pooled SMD 0.27; 95% CI [0.11, 0.44]; p=0.001) ( Figure 2). Pooled associations were not influenced by median cohort ICH volume (p=0.14) or age (p=0.68). Study quality did not influence pooled associations when risk of bias was assessed by the composite scale p=0.07 (Supplemental Figure 2A ^13^) but when assessed by the QUIPS tool the association between WBC and outcome was no longer statistically significant in studies with a low risk of bias (pooled SMD 0.22; 95% CI -0.04-0.48) (Supplemental Figure 2B). The pooled odds ratios (OR) of five studies ^49, 64, 72, 84, 89^ which reported an unadjusted OR was 1.06 (95% CI 1.02-1.10). There was no evidence of publication bias (Supplemental Figure 3A ^13^, Egger’s regression: p=0.23) and no outliers were identified. There was a high level of statistical heterogeneity (I ^2^=70%) between studies ( Figure 2).

In total, 18 studies ^30, 31, 43, 44, 52, 55, 61, 63, 64, 68, 69, 71, 74, 75, 83, 90, 94, 95^ examined the association between neutrophil count and clinical outcome. Three studies ^43, 55, 64^ of 629 participants measuring neutrophil count at admission were meta-analysed, one of which included a multivariate analysis ^64^. There was no association between neutrophil count and death or dependency at 90 days (pooled SMD 0.36; 95% CI [0.03, 0.75]; p=0.07; Supplemental Figure 4 ^13^). There was high statistical heterogeneity between studies (I ^2^=82%). A pooled OR of two studies ^31, 64^ that provided an adjusted OR showed no association between neutrophil count and outcome (OR 0.98, 95% CI 0.95-1.01).

Lymphocyte count was measured in 12 ^30, 31, 52, 61, 63, 68, 69, 71, 74, 75, 90, 94^ studies and NLR was measured in 10 ^30, 52, 63, 68, 69, 71, 74, 75, 96, 97^, but neither were meta-analysed because less than two studies reported mean/medians at 90-day mRS. Six (50%) studies found that lower lymphocyte counts were associated with worse outcome. Eight (80%) studies found an association between higher NLR and poor outcome by univariate analysis, which remained significant by multivariate analysis in six (75%) studies.

**Figure 3.**
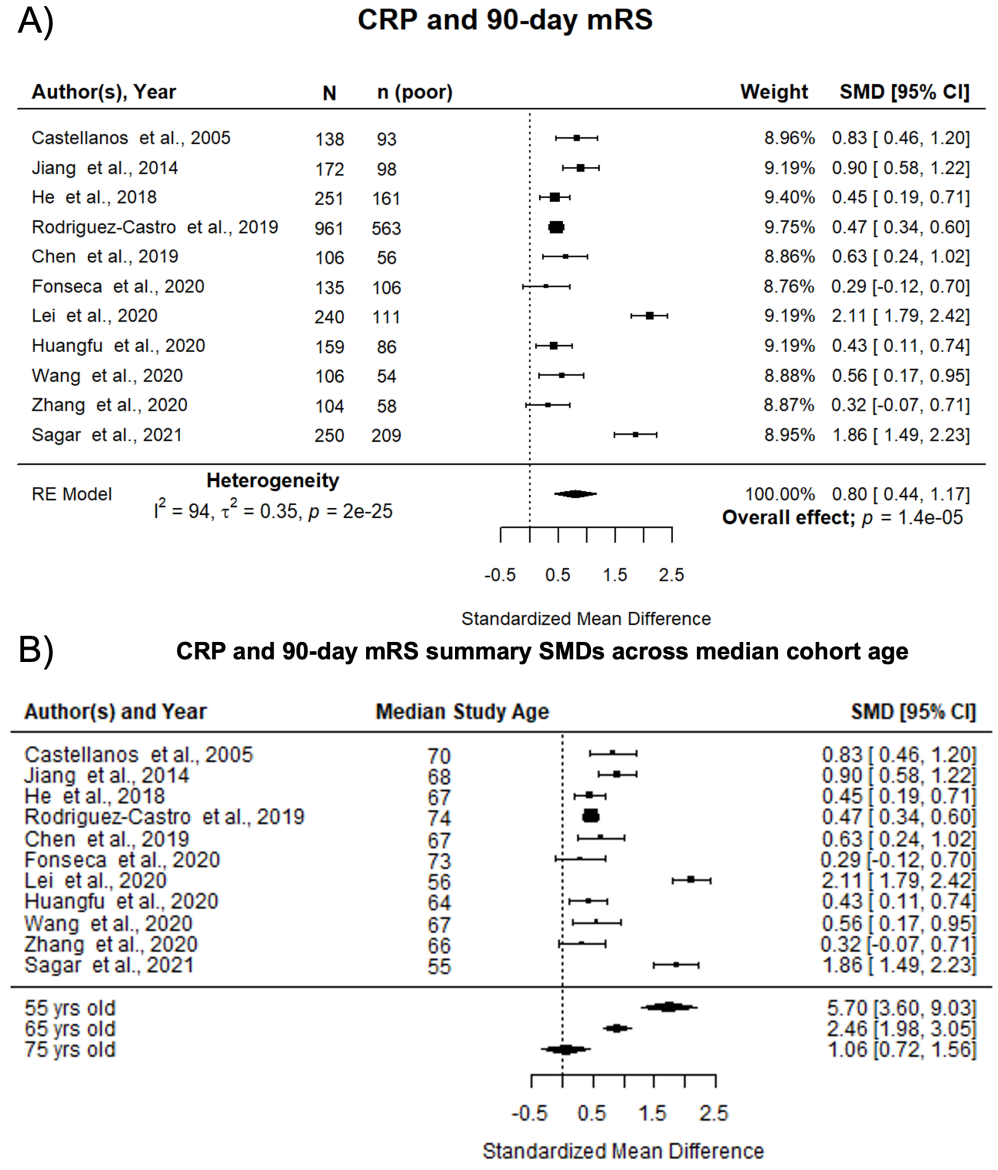
Pooled associations of CRP with death or dependency at 90 days after ICH. **A**) Forest plot of pooled associations of circulating CRP with death or dependency at 90 days. Poor outcome defined as mRS 3–6. **B**) Influence of age on the association of CRP with death or dependency at 90 days after ICH. Study cohorts with lower median cohort age have larger standardized mean differences between CRP and death or dependency at 90 days after ICH. CRP, C-reactive protein; ICH, intracerebral haemorrhage; mRS, modified Rankin Scale; SMD, standardised mean difference.

***Acute phase reactants.*** The most frequently measured acute phase protein was CRP in 31 studies ^31, 43, 45, 48, 49, 51, 54, 55, 57– 59, 64, 65, 70, 72, 73, 76– 78, 80, 84, 91, 92, 95, 98– 104^, including three that measured hypersensitive CRP ^54, 55, 95^. Of these, 11 studies of 2,622 participants which reported SMD were eligible for meta-analysis. Five of these studies (45%) included a multivariable analysis. Nine (82%) studies reported CRP at admission, the other two within 72 hours of ICH onset. Higher CRP was associated with death or dependency at 90 days (pooled SMD 0.80; 95% CI [0.44, 1.17]; p<0.0001) ( Figure 3A). Pooled associations were not influenced by median cohort ICH volume (p=0.43). However, with meta-regression, increasing median cohort age significantly reduced the association between high levels of CRP and worse outcome (beta -0.0841; p<0.001) ( Figure 3B). Study quality did not influence pooled associations when assessed both by the composite scale and by the QUIPS tool (Supplemental Figure 5A and 5B ^13^ The pooled adjusted OR of four studies ^31, 49, 64, 103^, three ^31, 49, 103^ of which had a low risk of bias according to QUIPS was 0.99 (95% CI 0.98-1.01). The pooled unadjusted OR of six studies ^31, 49, 84, 64, 72, 103^, four ^31, 49, 84, 103^ of which had a low risk of bias according to QUIPS was 1.00 (95% CI 0.98-1.01). There was no evidence of publication bias (Supplemental Figure 3B ^13^, Egger’s regression: p=0.84). Influence diagnostics identified one potential outlier ^64^, however summary effect sizes were similar when this study was removed and remained statistically significant (pooled SMD 0.67; 95% CI [0.39, 0.95]; p<0.001). We thus retained this study in the remaining analysis. There was a high level of statistical heterogeneity (I ^2^=94%) between studies.

**Figure 4.**
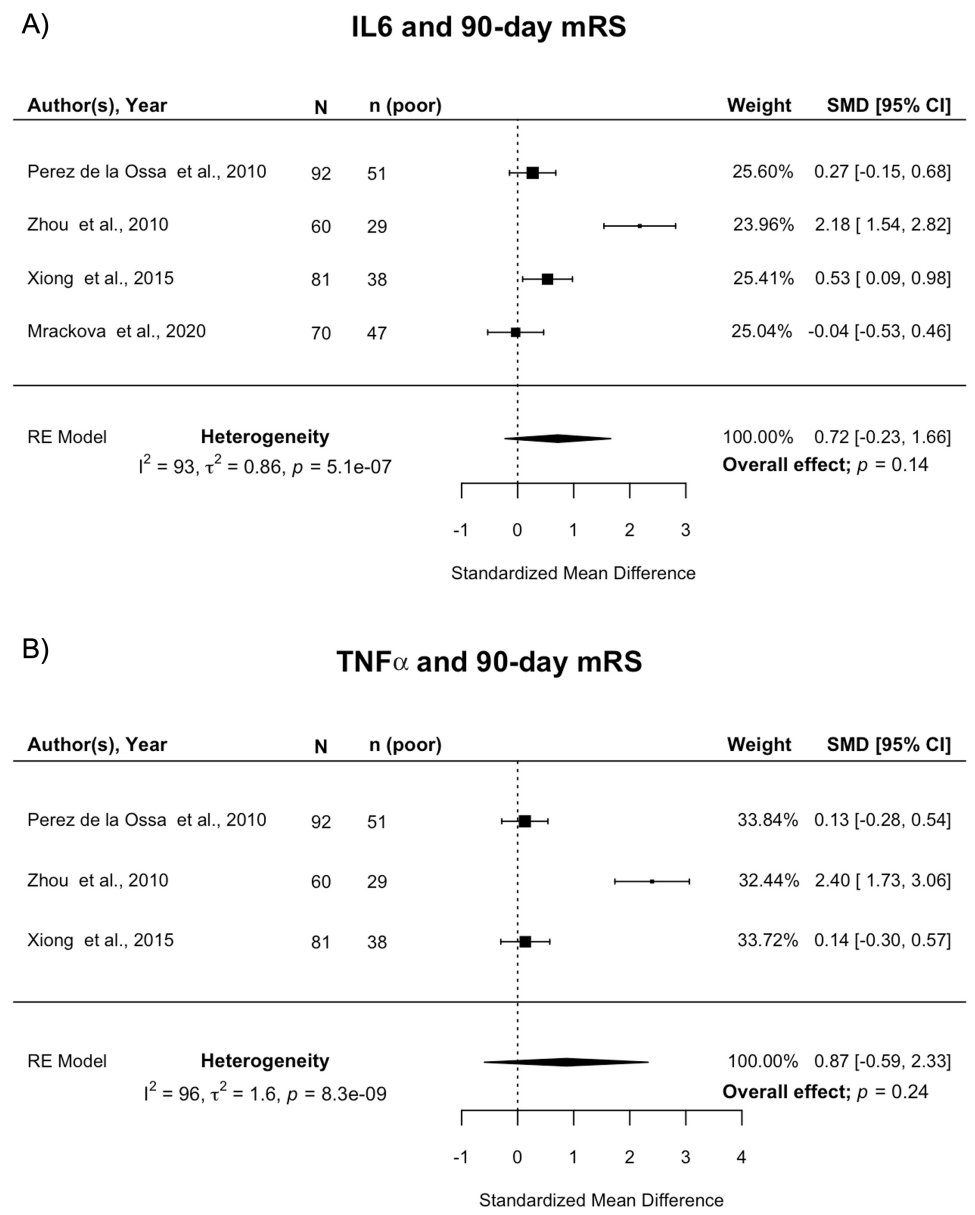
Forest plot of pooled associations of IL-6 and TNFα with death or dependency 90 days after ICH. **A**) Pooled association of circulating IL-6 levels with death or dependency at 90 days. **B**) Pooled association of circulating TNFα levels with death or dependency at 90 days. Poor outcome defined as mRS 3–6. ICH, intracerebral haemorrhage; mRS, modified Rankin Scale; SMD, standardised mean difference.

A total of 16 studies ^24, 27, 30, 38, 40– 42, 48, 49, 51, 59, 63, 70, 77, 78, 92^ reported on fibrinogen and clinical outcome. Seven studies of 1,833 participants measuring fibrinogen upon admission were meta-analysed ^29, 43, 55, 58, 59, 78, 93^, of which one included a multivariable analysis ^43^. Higher fibrinogen levels were associated with death and dependency at 90 days (pooled SMD 0.32; 95% CI [0.04, 0.61]; p=0.025) (Supplemental Figure 6 ^13^, No outliers were identified by influence diagnostics, thus, sensitivity analysis was not required. There was a high level of statistical heterogeneity (I ^2^= 85%) between studies.

From three studies reporting on ferritin and clinical outcome ^29, 32, 105^, two studies of 155 participants measuring ferritin upon admission were included in a meta-analysis ^29, 32^. Neither of these studies included a multivariable analysis. There was no association between ferritin and death or dependency at 90 days (pooled SMD 1.59; 95% CI [-0.94, 4.13]; p=0.22) (Supplemental Figure 7 ^13^). There was a high level of statistical heterogeneity (I ^2^=98%) between studies.

***Cytokines/chemokines.*** Two proinflammatory cytokines were included in meta-analyses; IL-6 ^29, 33, 72, 86, 93, 101, 106^ and TNFα ^29, 33, 86, 93, 101, 107^. Four studies ^29, 72, 86, 93^ of 303 participants reporting on IL-6 and 90-day mRS were meta-analysed, two of which included a multivariable analysis ^72, 86^. There was no association between IL-6 and death or dependency at 90 days (pooled SMD 0.72; 95% CI [-0.23, 1.66]; p=0.14) ( Figure 4A). Influence diagnostics identified one potential outlier ^93^. However, pooled estimates remained non-significant when this study was removed (pooled SMD 0.27; 95% CI [-0.03, 0.57]; p=0.08). There was a high level of statistical heterogeneity (I ^2^=93%) between studies. One study ^72^ reported an unadjusted OR of the association between IL-6 and outcome (OR 0.98 95% CI 0.95-1.02) and one other study reported an adjusted OR of the association (OR 0.90 95% CI 0.72-1.12) ^86^. Three studies of 233 participants reporting on TNFα ^29, 86, 93^ and 90-day mRS were meta-analysed, none of which included a multivariable analysis. There was no association between TNFα and death or dependency at 90 days (pooled SMD 0.87; 95% CI [-0.59, 2.33]; p=0.24) ( Figure 4B). There was a high level of statistical heterogeneity (I ^2^=96%) between studies.

Overall, 11 other cytokines were measured in 10 studies ^33, 34, 72, 101, 102, 104, 108– 111^ where high levels of seven cytokines were associated with worse clinical outcome, and this association was sustained in multi-variate analysis in four studies.

***Damage associated molecular patterns.*** Nine studies reported on S100B ^27, 72, 106, 112– 117^ and functional outcome. Four studies including 238 participants reporting on S100B upon admission and 90-day mRS were meta-analysed ^27, 72, 113, 117^, two of which included a multivariable analysis ^27, 72^. There was no association between S100B and death or dependency at 90 days (pooled SMD 0.55; 95% CI [-0.23, 1.32]; p=0.17) (Supplemental Figure 8 ^13^). Influence diagnostics identified one potential outlier ^117^. However, summary effect sizes were similar when this study was removed (pooled SMD 0.20; 95% CI [-0.09, 0.50]; p=0.18). There was a high level of statistical heterogeneity (I ^2^=87%) between studies.

**Figure 5.**
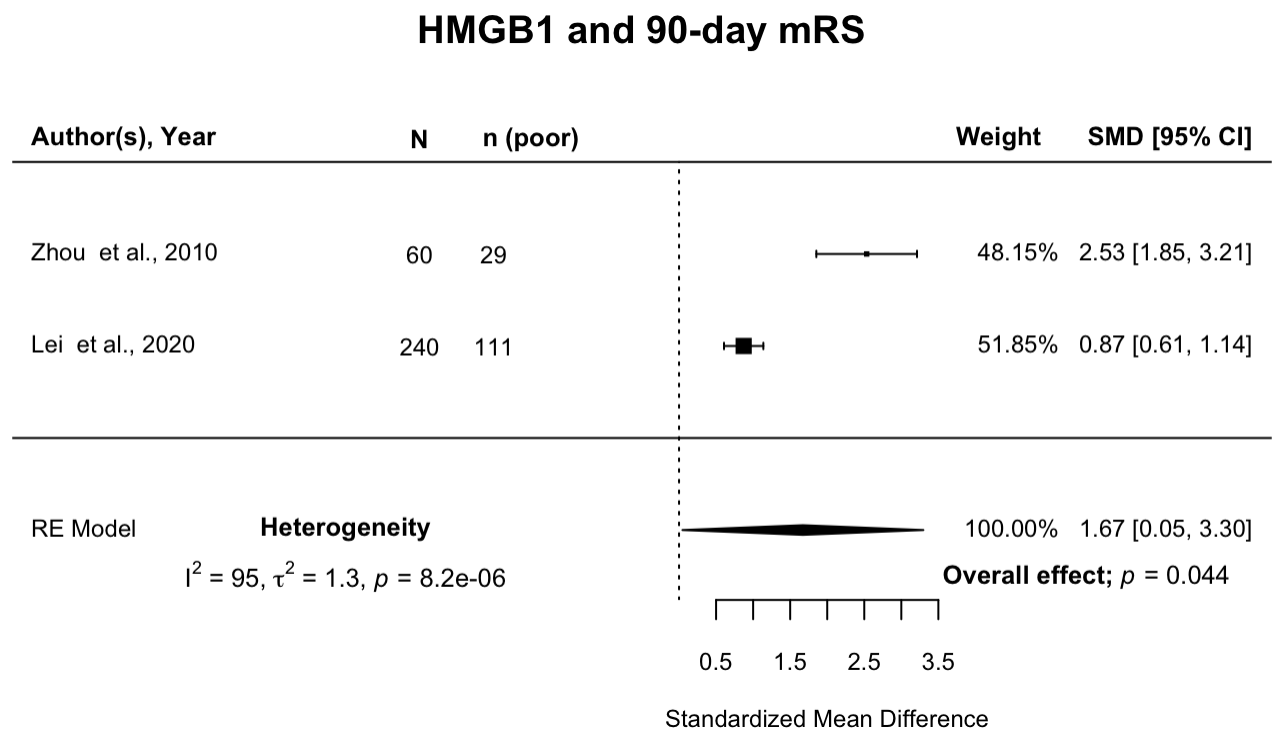
Forest plot of pooled association of HMGB1 with death or dependency at 90 days after ICH. Pooled association of circulating HMGB1 with death or dependency at 90 days. Poor outcome defined as mRS 3–6. HMGB1, high mobility group box protein 1; ICH, intracerebral haemorrhage; mRS, modified Rankin Scale; SMD, standardised mean difference.

Two studies ^64, 93^ of 300 participants reporting on HMGB1 upon admission and outcome were meta-analysed, neither of which included a multivariable analysis. Higher HMGB1 levels were associated with death or dependency at 90 days (pooled SMD 1.67; 95% CI [0.05, 3.30]; p=0.04) ( Figure 5). There was a high level of heterogeneity (I ^2^=95%) between studies.

***Tissue remodelling factors.*** Nine studies ^28, 34– 37, 72, 103, 118, 119^ reported on MMP-9 and clinical outcome, six of which found no association with outcome ^34, 36, 37, 72, 104, 119^. An additional six MMPs and two TIMPs were also reported by five of these studies ^28, 34, 36, 37, 119^ and no consistent association with clinical outcome was found. Meta-analysis was not performed because only single studies reported both biomarker concentrations and mRS at 90-days for each analyte.

***Adhesion molecules.*** Eight adhesion molecules were reported alongside clinical outcome in seven studies ^28, 33, 65, 66, 103, 120, 121^. However, as no single molecule was reported with 90-day mRS more than once, meta-analysis was not performed. Higher levels of four adhesion molecules; sCD40L, ICAM1, Selectin-E, Selectin-P, were associated with worse outcome in three studies ^33, 65, 103^. Three molecules; VCAM1, VEGF, Tim-3, showed no association in four studies ^28, 33, 66, 121^. Lower amounts of CD163 were associated with poor outcome in one study ^120^.

### Narrative synthesis of the association between inflammatory biomarkers and both PHO and outcome

Overall, 11 studies of 1,001 participants measured both PHO and clinical outcome. We did not find any biomarker that was consistently associated with both PHO and clinical outcome in more than one study and thus no meta-analysis could be performed. In one study of 116 participants, higher levels of IL-6, TNFα and ICAM1 were associated with larger PHO volumes and worse clinical outcomes in univariable analyses ^33^. In individual studies, S100B ^27^ and ferritin ^29^ were positively associated with both PHO and outcome in univariable analyses only. In one study (n=57) higher levels of MMP-9 were associated with larger PHO volumes in adults with supratentorial deep ICH and worse clinical outcomes at days 3–6 after ICH onset in univariable analyses ^35^. Studies of lymphocyte counts and NLR and their associations with PHO and outcome reported inconsistent findings ^30, 31^.

## Discussion

This systematic review and meta-analysis is the first to assess the relationship between inflammatory biomarkers and both PHO and outcome following ICH. From 93 studies examining the relationship between 50 biomarkers and functional outcome after ICH, we found that higher circulating levels of two biomarkers; fibrinogen and HMGB1, were associated with death or dependency at 90 days after ICH. A higher level of CRP seemed to be associated with death or dependency after ICH but when odds ratios were meta-analysed, this association did not remain. A higher level of WBC also seemed to be associated with death or dependency but this association did not remain in a pooled subgroup analysis of studies with a low risk of bias. We did not find an association between neutrophil count, ferritin, IL-6, TNFα or S100B and clinical outcome. We did not find any biomarker that was associated with both PHO and outcome in more than one study. The methodological heterogeneity between studies of inflammatory biomarkers and PHO precluded meta-analysis. However, we have narratively synthesised the findings from all published studies investigating the relationship between circulating inflammatory mediators and PHO

MMP-9 was the most frequently assessed biomarker in studies of PHO. It may be associated with PHO after ICH, but studies have been small with inconsistent findings and rarely controlled for important covariates such as ICH volume, which may act as confounders. There was heterogeneity in the timepoint of biomarker measurement which may also contribute to inconsistent findings since the level of a serum biomarker will vary in relation to the time of measurement after ICH onset. As MMPs have a role in the breakdown of the blood brain barrier, and contribute to vasogenic and cytotoxic PHO ^122, 123^, we believe this merits further investigation. Currently, these results are hypothesis generating and require replication in larger studies using a prespecified scanning protocol for the measurement of PHO.

A key finding from this study is the association between higher circulating HMGB1 levels and poorer outcome after ICH. HMGB1 is released from necrotic and inflammatory cells and stimulates DAMP receptors to amplify release of pro-inflammatory cytokines and recruit peripheral immune cells ^124, 125^. Its’ activity is increased after ICH in humans and is associated with the development of PHO ^126^. Since inhibiting HMGB1 improves outcome in animal models of ICH ^127, 128^ it may be a potential therapeutic target that merits further investigation.

Additionally we observed that higher levels of fibrinogen are associated with poor outcome and this is consistent with studies of ischemic stroke that have found similar associations ^129^, CRP may be associated with poor outcome after ICH ^130^, but we did not find a consistent association.

Although there seemed to be an association between WBC and outcome, the association no longer remained significant in a subgroup analysis of three studies (total sample size =304) with a low risk of bias. This may because we lacked power. This requires further exploration in larger prospective ICH cohorts.

These findings support the role of inflammation in ICH but do not provide information on the exact inflammatory pathways engaged or lead to potential therapeutic targets. Unlike HMGB1, which has been shown in preclinical studies to be specifically neutralised by anti-HMGB1 monoclonal antibodies ^128^, CRP is a non-specific marker of inflammation. For example, CRP is produced in the acute phase of most forms of inflammation and is thus non-specific to the inflammatory response to ICH. We believe that the analysis of more specific inflammatory mediators is required in order to gain a deeper understanding of the immune response to ICH and identify potential therapeutic targets. Of note however, the association of CRP with poor outcome is stronger as the median age of the cohort decreases. Whilst this could be explained by CRP biology as background serum levels of CRP increase during ageing ^131^, it could also be linked to confounders such as patient selection, since studies with lower median cohort ages had higher risk of bias scores.

Our meta-analysis did not find an association between circulating levels of either IL-6 or TNFα with death or dependency at 90 days. These cytokines stimulate the expression of acute phase proteins, such as CRP, and are thought to play detrimental roles in the pathogenesis of ICH ^8, 132^. Several factors may explain why we did not find an association: only four studies were included in these meta-analyses, the time point of biomarker measurement in relation to ICH onset varied, and peripheral circulating levels of these cytokines may not be comparable to concentrations in the brain. Moreover, the majority of studies only reported biomarker measurements upon admission or within 24hr. It is thus difficult to establish if these biomarker levels are reflective of the baseline physiology of individuals at risk of developing worse outcome or are directly caused by the onset of ICH itself. Longitudinal, serial blood sampling would assist in measuring the effect of ICH on systemic inflammatory levels independently of baseline levels.

We did not find any biomarker that was associated with both PHO and outcome in more than one study. Future studies should aim to identify if any biomarker is associated with both, since such a biomarker could either be a prognostic marker after ICH or act as an inflammatory mediator which would be more likely to be a potential therapeutic target. Since both animal studies and human studies (of brain tissue and serum) suggest the involvement of inflammatory pathways such as IL-1, HMGB-1, TNF in the perihaematomal region ^133^ and the development of PHO ^126^, and PHO itself is associated with outcome after ICH ^134, 135^, it is plausible that one or more such biomarkers could be involved in the inflammatory response in the brain following ICH.’ Modulating the neuroinflammatory response after ICH, for example by using an IL-1 receptor antagonist (NCT04834388) may therefore hold promise.

Our review is strengthened by a comprehensive search strategy of historic and contemporary literature without language or publication date restrictions. We did not limit our search strategy based on *a priori* knowledge of pre-specified biomarkers, and we identified many studies that were not considered in a recent review of the association between biomarkers and prognosis after ICH ^136^. We believe this is the first systematic review to assess the relationship between inflammatory biomarkers and both PHO and functional outcome after ICH. Critical appraisal of studies was determined by two independent reviewers and most meta-analyses were not affected by publication bias.

This study has some limitations. Our risk of bias assessment used a summed score, which may not fully reflect the degree of bias in certain studies. We encountered a high level of heterogeneity between studies in relation to biomarker measurement and PHO assessment, which precluded meta-analyses of some biomarkers. Given the heterogeneity and small numbers of studies of some biomarkers, we were unable to use meta-regression to determine the influence of variables such as ICH volume ^137^ on the association of all biomarkers with clinical outcome. None of the studies included in meta-analysis reported whether the biomarker helped to improve prediction of prognosis after ICH when compared or added to existing prognostic scores for ICH and this should be examined in future studies. We were unable to explore how variation in PHO measurement between studies affects any association between PHO and prognosis after ICH but this has been discussed elsewhere ^134, 135^.

## Conclusions

Higher levels of CRP, fibrinogen and HMGB1 were associated with worse outcome after ICH. Future prospective studies should prioritise the investigation of specific inflammatory mediators (such as HMGB1, cytokines, DAMPs and adhesion molecules) and adjust for key covariates such as ICH severity to better understand the pathophysiology of PHO and inflammation after ICH. This may reveal novel biomarkers, identify potential therapeutic targets and give better insights into certain immune-related post-ICH sequelae, such as infection.

## Ethics approval

An ethics statement is not applicable as this study is exclusively based on published literature.

## Data Availability

All data underlying the results are available as part of the article and no additional source data are required. Figshare: Supplementary Material to Kirby *et al.*, 2023_Association between circulating inflammatory biomarkers and functional outcome or perihaematomal oedema after ICH. https://doi.org/10.6084/m9.figshare.21995675 ^13^. This dataset contains the following *Extended data*: Supplementary Material Data are available under the terms of the Creative Commons Attribution 4.0 International license (CC-BY 4.0). Analysis code available from: https://github.com/Jack-Barrington/ICH_Biomarkers_Meta_Analysis Archived analysis code at time of publication: https://doi.org/10.5281/zenodo.7732800 ^26^. The Github repository contains: Formatted datasheet containing all meta-analysis (Clean_data.rds) Code to reproduce meta-analysis (WBC_Walkthrough.rmd) Data are available under the terms of the Creative Commons Zero "No rights reserved" data waiver (CC0 1.0 Public domain dedication). Figshare: PRISMA checklist for ‘Association between circulating inflammatory biomarkers and functional outcome or perihaematomal oedema after ICH: a systematic review & meta-analysis’. https://doi.org/10.6084/m9.figshare.22312042 ^12^. Data are available under the terms of the Creative Commons Attribution 4.0 International license (CC-BY 4.0).
