# Supplementary material for: Association between circulating inflammatory biomarkers and functional outcome or perihaematomal oedema after ICH: a systematic review & meta-analysis
Source: Wellcome Open Res. 2023 Nov 21;8:239. Originally published 2023 Jun 12. [Version 2] doi: 10.12688/wellcomeopenres.19187.2 (PMC10687391; doi:10.12688/wellcomeopenres.19187.2)
Supplement: Peer review history

# Reviewer report: Reviewer response for version 2

**DOI:** 10.21956/wellcomeopenres.22697.r70300

**Published:** 2023-12-28

**Authors:** Jessica R Magid-Bernstein

**Affiliations:** 1 Department of Neurology, Yale University, New Haven, Connecticut, USA

The authors appropriately addressed the one concern I had about the article.

Are the rationale for, and objectives of, the Systematic Review clearly stated?

Yes

Is the statistical analysis and its interpretation appropriate?

I cannot comment. A qualified statistician is required.

Are sufficient details of the methods and analysis provided to allow replication by others?

Yes

Are the conclusions drawn adequately supported by the results presented in the review?

Yes

Reviewer Expertise:

Inflammation in intracerebral hemorrhage

I confirm that I have read this submission and believe that I have an appropriate level of expertise to confirm that it is of an acceptable scientific standard.

# Reviewer report: Reviewer response for version 2

**DOI:** 10.21956/wellcomeopenres.22697.r70301

**Published:** 2023-12-07

**Authors:** Jian Wang, Qinfeng Peng

**Affiliations:** 1 Department of Pain Medicine, Department of Human Anatomy, Zhengzhou University, Zhengzhou, Henan, China; 2 Department of Human Anatomy, College of Basic Medicine, Zhengzhou University, Zhengzhou, Henan, China

The authors have addressed our concerns. We do not have other critiques.

Are the rationale for, and objectives of, the Systematic Review clearly stated?

Yes

Is the statistical analysis and its interpretation appropriate?

I cannot comment. A qualified statistician is required.

Are sufficient details of the methods and analysis provided to allow replication by others?

Partly

Are the conclusions drawn adequately supported by the results presented in the review?

Partly

Reviewer Expertise:

NA

We confirm that we have read this submission and believe that we have an appropriate level of expertise to confirm that it is of an acceptable scientific standard.

# Reviewer report: Reviewer response for version 2

**DOI:** 10.21956/wellcomeopenres.22697.r70302

**Published:** 2023-11-30

**Authors:** Felipe A. Montellano

**Affiliations:** 1 Department of Neurology, University Hospital Würzburg, Würzburg, Germany; 2 Institute of Clinical Epidemiology and Biometry, University of Würzburg, Würzburg, Germany

The authors have satisfactorily addressed my remarks in the revised version of the manuscript. I congratulate the authors once again for the thoroughness of their work. I have no further comments.

Are the rationale for, and objectives of, the Systematic Review clearly stated?

Yes

Is the statistical analysis and its interpretation appropriate?

Partly

Are sufficient details of the methods and analysis provided to allow replication by others?

Yes

Are the conclusions drawn adequately supported by the results presented in the review?

Partly

Reviewer Expertise:

Stroke, prognosis research

I confirm that I have read this submission and believe that I have an appropriate level of expertise to confirm that it is of an acceptable scientific standard.

# Reviewer report: Reviewer response for version 2

**DOI:** 10.21956/wellcomeopenres.22697.r70303

**Published:** 2023-11-29

**Authors:** Sarah Marchina

**Affiliations:** 1 Department of Neurology, Stroke Division, Beth Israel Deaconess Medical Center, Harvard Medical School, Boston, Massachusetts, USA

I would like to thank the authors for addressing my comments, I am satisfied with the responses. I have no further questions on the revised manuscript.

Are the rationale for, and objectives of, the Systematic Review clearly stated?

Yes

Is the statistical analysis and its interpretation appropriate?

Yes

Are sufficient details of the methods and analysis provided to allow replication by others?

Yes

Are the conclusions drawn adequately supported by the results presented in the review?

Yes

Reviewer Expertise:

Intracerebral hemorrhage, stroke,

I confirm that I have read this submission and believe that I have an appropriate level of expertise to confirm that it is of an acceptable scientific standard.

# Reviewer report: Reviewer response for version 1

**DOI:** 10.21956/wellcomeopenres.21268.r64231

**Published:** 2023-09-05

**Authors:** Jessica R Magid-Bernstein

**Affiliations:** 1 Department of Neurology, Yale University, New Haven, Connecticut, USA

The authors present a very comprehensive systematic review and meta analysis investigating inflammatory markers and their relationship with both imaging markers of inflammation and outcome after ICH. Overall the review is very well done and well written, and I have very few comments. 

 The only minor edit I would suggest is that in the Narrative synthesis of the association between inflammatory biomarkers and perihematomal edema section, the authors discuss a few studies that investigated the association between certain markers and midline shift, but this is the first time MLS is mentioned. In the Introduction and Methods sections they only discuss PHO as an imaging marker of inflammation. I think it could be reasonable to use midline shift as an imaging marker of inflammation, especially if they believe/state that the midline shift is due to PHO, but this is not discussed. If these studies investigated MLS as an imaging marker of neuroinflammation since it was due to PHO, then it would make sense to include these studies but the authors should make this clear.

Are the rationale for, and objectives of, the Systematic Review clearly stated?

Yes

Is the statistical analysis and its interpretation appropriate?

I cannot comment. A qualified statistician is required.

Are sufficient details of the methods and analysis provided to allow replication by others?

Yes

Are the conclusions drawn adequately supported by the results presented in the review?

Yes

Reviewer Expertise:

Inflammation in intracerebral hemorrhage

I confirm that I have read this submission and believe that I have an appropriate level of expertise to confirm that it is of an acceptable scientific standard, however I have significant reservations, as outlined above.

# Response

**Published:** 2023-11-15

**Authors:** Caoimhe Kirby

**Affiliations:** DIIIRM, The University of Manchester, Manchester, England, UK

**The authors present a very comprehensive systematic review and meta analysis investigating inflammatory markers and their relationship with both imaging markers of inflammation and outcome after ICH. Overall the review is very well done and well written, and I have very few comments.** *Thank you* **The only minor edit I would suggest is that in the Narrative synthesis of the association between inflammatory biomarkers and perihematomal edema section, the authors discuss a few studies that investigated the association between certain markers and midline shift, but this is the first time MLS is mentioned. In the Introduction and Methods sections they only discuss PHO as an imaging marker of inflammation. I think it could be reasonable to use midline shift as an imaging marker of inflammation, especially if they believe/state that the midline shift is due to PHO, but this is not discussed. If these studies investigated MLS as an imaging marker of neuroinflammation since it was due to PHO, then it would make sense to include these studies but the authors should make this clear.**  

 Response: Thank you for this important point. We recently completed a systematic review and meta-analysis of studies examining the relationship between PHO and outcome after spontaneous ICH (Cliteur et al. 2023; 10.1177/23969873231157884) and did not include studies which used midline shift as their sole measure of PHO since midline shift may be due to ICH volume rather than PHO volume.  In view of this, we have removed the single study which used midline shift as its sole measure of PHO from Table 1, and also commented on this in the text: ‘In  total,  16  studies of  1,299  participants  assessed  the  relationship  between  21  inflammatory  biomarkers  and  PHO  (14 by   CT,   two   by   MRI)   (Table   1;   participant characteristics listed in Supplemental Table 2), of which we excluded one study ^28^ which assessed PHO using midline shift only’.

# Reviewer report: Reviewer response for version 1

**DOI:** 10.21956/wellcomeopenres.21268.r64212

**Published:** 2023-08-23

**Authors:** Sarah Marchina

**Affiliations:** 1 Department of Neurology, Stroke Division, Beth Israel Deaconess Medical Center, Harvard Medical School, Boston, Massachusetts, USA

I Summary

 Thank you for giving me the opportunity to comment on the manuscript “Association between circulating inflammatory biomarkers and functional outcome or perihaematomal oedema after ICH: a systematic review & meta-analysis”. The authors hypothesize that inflammatory biomarkers in peripheral blood may be associated with clinical outcome following ICH, as well as perihaematomal oedema (PHO). The latter association was assessed in 16 studies while the former relation between biomarkers and clinical outcome was examined in 93 studies. The authors came to the conclusion that higher levels of WBC, CRP, fibrinogen and HMGB1 were associated with worse outcome after ICH.

 II Strengths

 Overall, this is a very comprehensive and excellently conducted meta-analysis which will hopefully act as an inspiration/stepping stone for further research into this important topic.

 The study follows the PRISMA guidelines. The introduction clearly states what the aims of the study are and why this project was undertaken, followed by appropriate methods to achieve that aim. The results are well presented with forest plots, tables and additional material in the supplementary data.

 As a reviewer, I have a few suggestions for consideration to the authors. I divided them into the section of the manuscript rather than major/minor points.

 III Suggestions to improve the manuscript

 Methods

- How was the de-duplication done? This would be a small but helpful information for readers and especially researchers who would like to embark on conducting a meta-analysis themselves.
- I am missing a paragraph describing the outcome measures (and potentially timepoints) used in the studies. Since this study looks at associations between biomarkers and outcome, this information is crucial. Please add this.
- Using standardized mean differences (SMD) in this analysis seems very appropriate to me. But it would be important to give the reader a brief explanation as to why you picked this measure. Also, mention which SMD you used (Cohen’s d, Hedges, Glass’ delta etc., see Andrade, J Clin Psychiatry 2020)
- Please provide a sentence or two as to why you chose the restricted maximum likelihood random effects model over other very commonly used models such as the DerSimonian-Laird model.
- Risk of bias assessment: The authors developed a new scale to assess the quality of the included studies. Considering the large number of papers included in this study it is understandable that the authors would aim for a simple and short form of assessment. That being said, it is important to assess all aspects of a study and it seems like there are no questions pertaining to statistical analyses and/or confounding in the self-developed questionnaire. Please provide a reasoning for the decision to use a self-developed scale (instead of one of the existing ones such as STROBE-ME or QUIPS in their entirety) and how you picked the final items for your scale. Also, add the initials of the authors who performed the risk of bias and explain how you resolved potential disagreements.

Results:

- On page 9 it would be helpful for the reader to mention the term meta-regression in the text
- You report after each meta-analysis that the heterogeneity between studies was high. It would be helpful to have the I ^2^ in a bracket provided to the reader (the actual number).
- You mention the sensitivity analysis in the methods section, but I don’t see any results to this effect for any of the meta-analyses or any mention of it except of for the fibrinogen group. Was this done for all meta-analyses which had more than 3 studies included?
- In the paragraph about Cytokines (page 11), you provide the pooled estimates for the analysis and for the analysis sans outlier and mention they were similar. It seems that they were not exactly similar, but they both stayed non-significant.
- In the same chapter as above on page 11 you mention 11 other Cytokines. It was unclear to me as to why there wasn’t any meta-analysis on this – especially since you mention a significant association with outcome. Could you provide some clarification to this paragraph?
- Funnel plots in the supplementary material:
  - Every single funnel plot figure caption says: “Symmetrical distribution indicates a low probability of publication bias”, however, Figure 4, 7 and 8 don’t look very symmetrical which is probably why you reanalyzed without the outlier. I suggest to rephrase the figure caption for those figures.

Discussion:

- In the first sentence of the discussion you talk about “all” biomarkers. Clarify whether you mean all existing ones to date (in all literature) or all you found with regards to the question you asked.
- It seems that PHO and biomarkers are somewhat treated as separate entities in this manuscript, however, both are interrelated and part of the secondary inflammatory response to the ICH. It would be helpful to work out/clarify this relationship a bit more in the discussion and/or conclusion and try to elaborate how they both relate to outcome.
- The conclusion is very general. As reader I would be curious to know – after this extensive presentation and summary of literature – how this all translates into practice. What would we expect *if* there was a clear relationship between biomarker and outcome or a clear relationship between PHO and biomarkers/outcome. What could potential therapeutic targets be or how could a potential therapy look like? How would this change clinical practice, if at all?

Are the rationale for, and objectives of, the Systematic Review clearly stated?

Yes

Is the statistical analysis and its interpretation appropriate?

Yes

Are sufficient details of the methods and analysis provided to allow replication by others?

Yes

Are the conclusions drawn adequately supported by the results presented in the review?

Yes

Reviewer Expertise:

Intracerebral hemorrhage, stroke,

I confirm that I have read this submission and believe that I have an appropriate level of expertise to confirm that it is of an acceptable scientific standard, however I have significant reservations, as outlined above.

## References

[1] : Mean Difference, Standardized Mean Difference (SMD), and Their Use in Meta-Analysis: As Simple as It Gets. *J Clin Psychiatry* .2020;81(5) : 10.4088/JCP.20f13681 10.4088/JCP.20f13681 32965803

# Response

**Published:** 2023-11-15

**Authors:** Caoimhe Kirby

**Affiliations:** DIIIRM, The University of Manchester, Manchester, England, UK

**Thank you for giving me the opportunity to comment on the manuscript “Association between circulating inflammatory biomarkers and functional outcome or perihaematomal oedema after ICH: a systematic review & meta-analysis”. The authors hypothesize that inflammatory biomarkers in peripheral blood may be associated with clinical outcome following ICH, as well as perihaematomal oedema (PHO). The latter association was assessed in 16 studies while the former relation between biomarkers and clinical outcome was examined in 93 studies. The authors came to the conclusion that higher levels of WBC, CRP, fibrinogen and HMGB1 were associated with worse outcome after ICH.** **Strengths** **Overall, this is a very comprehensive and excellently conducted meta-analysis which will hopefully act as an inspiration/stepping stone for further research into this important topic.** **The study follows the PRISMA guidelines. The introduction clearly states what the aims of the study are and why this project was undertaken, followed by appropriate methods to achieve that aim. The results are well presented with forest plots, tables and additional material in the supplementary data.** Thank you. **As a reviewer, I have a few suggestions for consideration to the authors. I divided them into the section of the manuscript rather than major/minor points.** **III Suggestions to improve the manuscript** **Methods** **How was the de-duplication done? This would be a small but helpful information for readers and especially researchers who would like to embark on conducting a meta-analysis themselves.**

 Response: Thank you for requesting this clarification. We have amended the methods: ‘After automated de-duplication in Covidence (covidence.org), three authors…’

**I am missing a paragraph describing the outcome measures (and potentially timepoints) used in the studies. Since this study looks at associations between biomarkers and outcome, this information is crucial. Please add this.**

 Response: Thank you for requesting this. All measures of outcome and time points are given in Supplemental Table 1.

**Using standardized mean differences (SMD) in this analysis seems very appropriate to me. But it would be important to give the reader a brief explanation as to why you picked this measure. Also, mention which SMD you used (Cohen’s d, Hedges, Glass’ delta etc., see Andrade, J Clin Psychiatry 2020)**

 Response: Thank you for requesting this clarification. We used the Hedges g for estimation of SMD because it considers sample size when calculating overall effect size and have now included this in the manuscript in the Methods section (meta-analysis).  We used SMD because it was the most commonly used outcome measure.

**Please provide a sentence or two as to why you chose the restricted maximum likelihood random effects model over other very commonly used models such as the DerSimonian-Laird model.**

 Response: Thank you for requesting this clarification. We chose the restricted maximum likelihood random effects model because it shows slightly less bias in simulations ( https://onlinelibrary.wiley.com/doi/10.1002/jrsm.1316 [onlinelibrary.wiley.com]), is easy to access through openly available R packages such as metafor, and computational burden was not a factor.  

**Risk of bias assessment: The authors developed a new scale to assess the quality of the included studies. Considering the large number of papers included in this study it is understandable that the authors would aim for a simple and short form of assessment. That being said, it is important to assess all aspects of a study and it seems like there are no questions pertaining to statistical analyses and/or confounding in the self-developed questionnaire. Please provide a reasoning for the decision to use a self-developed scale (instead of one of the existing ones such as STROBE-ME or QUIPS in their entirety) and how you picked the final items for your scale. Also, add the initials of the authors who performed the risk of bias and explain how you resolved potential disagreements**.

 Response: Thank you for requesting this clarification.  As per our response to a comment raised by reviewer 1, we used a self-developed scale as although the STROBE-ME guidelines, Newcastle Ottawa scale and REMARK guidelines cover aspects of assessment of risk of bias we did not think that any of these three guidelines covered all the aspects that should be included (for example – the STROBE-ME and Newcastle Ottawa scale checklists do not include any aspects specific to biomarker measurement, while the REMARK checklist seems to place less emphasis on participant selection in comparison to the others but includes specific information relevant to serum biomarker studies.)  We have now also rated each of the studies included in a meta-analysis using the QUIPS tool and included this information in the supplement. We have also made it clearer in the methods (data extraction section) that risk of bias was assessed by the same authors who extracted data from the studies (CK, JB or NS) and conflicts relating to RoB were assessed by the same arbitrator that arbitrated conflicts relating to data extraction (JL, FS).  We have amended the abstract to indicate that we assessed risk of bias using both a composite scale and the QUIPS tool (for those studies being meta-analysed).    

**Results:** **On page 9 it would be helpful for the reader to mention the term meta-regression in the text**

 Response: Thank you. We have added the term ‘meta-regression’ into the text.

**You report after each meta-analysis that the heterogeneity between studies was high. It would be helpful to have the I2 in a bracket provided to the reader (the actual number).**

 Response: Thank you for requesting this clarification. We have added the I ^2 ^statistic into each sentence in the results section which describes heterogeneity.

**You mention the sensitivity analysis in the methods section, but I don’t see any results to this effect for any of the meta-analyses or any mention of it except of for the fibrinogen group. Was this done for all meta-analyses which had more than 3 studies included?**

 Response: Thank you for this important comment. We only used a sensitivity analysis if we identified an outlier.  We have made this clearer in the methods (meta-analysis section) ‘Where  more  than  three  studies  were  included in   meta-analysis,   we   evaluated   outliers   both   graphically and  via  influence  diagnostics and if we identified an outlier, sensitivity  was  assessed by leave-one-out analysis.

**In the paragraph about Cytokines (page 11), you provide the pooled estimates for the analysis and for the analysis sans outlier and mention they were similar. It seems that they were not exactly similar, but they both stayed non-significant.**

 Response: Thank you. We have corrected this in the results section (cytokines). ‘However, pooled estimates remained non-significant when this study was removed…’

**In the same chapter as above on page 11 you mention 11 other Cytokines. It was unclear to me as to why there wasn’t any meta-analysis on this – especially since you mention a significant association with outcome. Could you provide some clarification to this paragraph?**

 Response: Thank you for requesting this clarification.  Apart from IL-6 and TNFa, no other cytokines were analysed with the same outcome measure in more than one study so no meta-analysis could be performed (Supplemental Table 1).

**Funnel plots in the supplementary material:** **Every single funnel plot figure caption says: “Symmetrical distribution indicates a low probability of publication bias”, however, Figure 4, 7 and 8 don’t look very symmetrical which is probably why you re-analyzed without the outlier. I suggest to rephrase the figure caption for those figures.**

 Response: Thank you for this important point. Since funnel plots should only be used when there are at least 10 studies included in the meta-analysis ( https://handbook-5-1.cochrane.org/chapter_10/10_4_3_1_recommendations_on_testing_for_funnel_plot_asymmetry.htm)  we have now removed the funnel plots for biomarkers with fewer than 10 studies (fibrinogen, IL-6, S100B). We have provided a new DOI for supplemental material.

**Discussion:** **In the first sentence of the discussion you talk about “all” biomarkers. Clarify whether you mean all existing ones to date (in all literature) or all you found with regards to the question you asked.**

 Response: Thank you for requesting this clarification. We meant all existing ones to date identified in the literature by our search strategy, but to simplify the wording we have removed the word ‘all’.

**It seems that PHO and biomarkers are somewhat treated as separate entities in this manuscript, however, both are interrelated and part of the secondary inflammatory response to the ICH. It would be helpful to work out/clarify this relationship a bit more in the discussion and/or conclusion and try to elaborate how they both relate to outcome.** **The conclusion is very general. As reader I would be curious to know – after this extensive presentation and summary of literature – how this all translates into practice. What would we expect if there was a clear relationship between biomarker and outcome or a clear relationship between PHO and biomarkers/outcome. What could potential therapeutic targets be or how could a potential therapy look like? How would this change clinical practice, if at all?**

 Response: Thank you for both these comments. We have added the following paragraph into the discussion: ‘Future  studies should aim to identify if any biomarker is associated with both, since  such  a biomarker could either be a prognostic marker after ICH or act as an  inflammatory  mediator  which would  be  more  likely to be a potential therapeutic target. Since both animal studies and human studies (of brain tissue and serum) suggest the involvement of inflammatory pathways such as IL-1, HMGB-1 and TNF in the perihaematomal region ^130^ and the development of PHO, ^123^ and PHO itself is associated with outcome after ICH, ^131,132^ it is plausible that one or more such biomarkers could be involved in the inflammatory response in the brain following ICH’ **.  **Modulating the neuroinflammatory response after ICH, for example by using an IL-1 receptor antagonist (NCT04834388) may therefore hold promise’.

# Reviewer report: Reviewer response for version 1

**DOI:** 10.21956/wellcomeopenres.21268.r62060

**Published:** 2023-08-14

**Authors:** Jian Wang, Qinfeng Peng

**Affiliations:** 1 Department of Pain Medicine, Department of Human Anatomy, Zhengzhou University, Zhengzhou, Henan, China; 2 Department of Human Anatomy, College of Basic Medicine, Zhengzhou University, Zhengzhou, Henan, China

**Association between circulating inflammatory biomarkers and functional outcome or perihaematomal oedema after ICH: a systematic review & meta-analysis (Peer review)**

 This study examined the effects of inflammatory biomarkers in peripheral blood on the prognosis of ICH and discussed relevant research on perihaematomal oedema (PHO). The study found that higher inflammatory markers, such as WBC count, CRP, fibrinogen, and HMGB1, are associated with poorer prognoses after intracerebral hemorrhage (ICH).

 Our assessment of this paper is as follows. Firstly, the research's aim is clear, and the background, scientific premise, main content, and table organization are clear and complete. Secondly, the authors thoroughly described the literature screening process, which was well-organized and concise. Additionally, the authors categorized inflammatory markers and analyzed the relationship between inflammatory markers and the prognosis of ICH from various perspectives, providing novel ideas for future research on inflammatory markers in ICH. While we cannot provide better recommendations on analytical and statistical methods, we do have a few suggestions to enhance the study's research design.

1. The authors selected 98 studies for meta-analysis and established criteria to exclude ineligible studies. However, it would be helpful if the authors explained their reasoning for establishing these criteria. Furthermore, it would be beneficial for the authors to ensure that the quality of the selected studies meets rigorous research standards.
2. In the Introduction, the authors point out that PHO is an imaging biomarker of the neuroinflammatory response. However, it's important to note that PHO is related to neuroinflammation and other secondary brain injury after ICH, including blood-brain damage ^[1]^. Furthermore, PHO can be divided into different stages, and neuroinflammation may lead to PHO development rather than PHO solely representing neuroinflammation ^[2]^.
3. The authors categorized inflammatory markers into six groups based on their biological properties: immune cells, cytokines/chemokines, tissue remodeling factors, damage-associated molecular patterns (DAMP), acute phase reactants, and adhesion molecules. However, the classification criteria may need reconsidering because some categories, such as acute phase reactants, may overlap. The authors should justify their classification system.
4. The authors studied the connection between inflammatory markers and the prognosis of ICH, but they did not examine the relationship between PHO and ICH prognosis. The issue may be that various studies used different methods to measure PHO, so the differences in research approaches require clarification and summarization. The authors should investigate whether variations in research methods for measuring PHO could impact the prognosis of ICH.
5. In their study, the authors found that MMP-9 showed a positive correlation with the volume of PHO and that MMP-2 and MMP-8 were positively correlated with the absolute volume of PHO. However, another study revealed that on the 7th day after ICH, MMP-2 had a negative correlation with PHO volume ^[3]^. It is worth noting that the authors did not consider changes in the inflammatory response at different time points, which could affect the interpretation of the results. It is expected that there would be differences in each inflammatory marker at various stages of ICH.
6. According to the study, there is a connection between the volume of PHO and IL-10. However, previous research has stated that there is a negative relationship between PHO volume and IL-10 ^[4]^. Another study found that higher levels of IL-10 in the blood are linked to a favorable prognosis 90 days after ICH. Additionally, administering IL-10 through the nose has decreased PHO and enhanced functional outcomes of mice with ICH ^[5]^. As a result, the authors need to approach their findings with caution.

Are the rationale for, and objectives of, the Systematic Review clearly stated?

Yes

Is the statistical analysis and its interpretation appropriate?

I cannot comment. A qualified statistician is required.

Are sufficient details of the methods and analysis provided to allow replication by others?

Partly

Are the conclusions drawn adequately supported by the results presented in the review?

Partly

Reviewer Expertise:

Intracerebral hemorrhage, Cerebrovascular disease

We confirm that we have read this submission and believe that we have an appropriate level of expertise to confirm that it is of an acceptable scientific standard, however we have significant reservations, as outlined above.

## References

[1] : Profiling of Blood-Brain Barrier Disruption in Mouse Intracerebral Hemorrhage Models: Collagenase Injection vs. Autologous Arterial Whole Blood Infusion. *Front Cell Neurosci* .2021;15: 10.3389/fncel.2021.699736 699736 10.3389/fncel.2021.699736 34512265 PMC8427528

[2] : Targeting secondary injury in intracerebral haemorrhage--perihaematomal oedema. *Nat Rev Neurol* .2015;11(2) : 10.1038/nrneurol.2014.264 111-22 10.1038/nrneurol.2014.264 25623787

[3] : Timing of serum active MMP-9 and MMP-2 levels in acute and subacute phases after spontaneous intracerebral hemorrhage. *Acta Neurochir Suppl* .2010;106: 10.1007/978-3-211-98811-4_24 137-40 10.1007/978-3-211-98811-4_24 19812936

[4] : Expressions of serum inflammatory cytokines and their relationship with cerebral edema in patients with acute basal ganglia hemorrhage. *Eur Rev Med Pharmacol Sci* .2016;20(13) :2868-71 27424987

[5] : Immune changes in peripheral blood and hematoma of patients with intracerebral hemorrhage. *FASEB J* .2020;34(2) : 10.1096/fj.201902478R 2774-2791 10.1096/fj.201902478R 31912591

[6] : Microglia-derived interleukin-10 accelerates post-intracerebral hemorrhage hematoma clearance by regulating CD36. *Brain Behav Immun* .2021;94: 10.1016/j.bbi.2021.02.001 437-457 10.1016/j.bbi.2021.02.001 33588074 PMC8058329

# Response

**Published:** 2023-11-15

**Authors:** Caoimhe Kirby

**Affiliations:** DIIIRM, The University of Manchester, Manchester, England, UK

**This study examined the effects of inflammatory biomarkers in peripheral blood on the prognosis of ICH and discussed relevant research on perihaematomal oedema (PHO). The study found that higher inflammatory markers, such as WBC count, CRP, fibrinogen, and HMGB1, are associated with poorer prognoses after intracerebral hemorrhage (ICH).** **Our assessment of this paper is as follows. Firstly, the research's aim is clear, and the background, scientific premise, main content, and table organization are clear and complete. Secondly, the authors thoroughly described the literature screening process, which was well-organized and concise. Additionally, the authors categorized inflammatory markers and analyzed the relationship between inflammatory markers and the prognosis of ICH from various perspectives, providing novel ideas for future research on inflammatory markers in ICH. While we cannot provide better recommendations on analytical and statistical methods, we do have a few suggestions to enhance the study's research design.** **The authors selected 98 studies for meta-analysis and established criteria to exclude ineligible studies. However, it would be helpful if the authors explained their reasoning for establishing these criteria. Furthermore, it would be beneficial for the authors to ensure that the quality of the selected studies meets rigorous research standards.**

 Thank you for requesting this clarification. We list the criteria for determining which studies were deemed ineligible in Figure 1, with the most frequent five reasons that studies were deemed ineligible being:

1. Studies were published as conference abstracts only
2. Studies were narrative or systematic reviews
3. No relationship between an inflammatory biomarker and either PHO or clinical outcome was measured.
4. Patients treated with a surgical intervention included in the analysis
5. Mixed causes of ICH or stroke included in the analysis.

We excluded studies that included patients treated with a surgical intervention (when we were unable to separate in the analysis those treated with surgery from those not treated with surgery) since surgery may affect both the biomarker (for example, CRP may rise with surgery) and PHO or outcome, thereby acting as a confounder. We excluded studies which had a mixed cohort (for example including patients with ischaemic stroke or patients who had ICH due to a structural cause such as an aneurysm or a tumour) when we were unable to separate those with spontaneous ICH from the rest in the analysis.  The inflammatory response after spontaneous ICH may differ from those with ICH or stroke due to another cause. We agree that it is important to ensure that the quality of the selected studies meets rigorous research standards and have therefore re-rated all studies included in meta-analyses using the QUIPS tool (see response to reviewer 1) whilst also retaining our risk of bias assessment using the composite scale.

**In the Introduction, the authors point out that PHO is an imaging biomarker of the neuroinflammatory response. However, it's important to note that PHO is related to neuroinflammation and other secondary brain injury after ICH, including blood-brain damage[1]. Furthermore, PHO can be divided into different stages, and neuroinflammation may lead to PHO development rather than PHO solely representing neuroinflammation[2].**

 Response: Thank you for raising this point. We have added in text to reflect this in the introduction - Perihaematomal oedema (PHO) is thought to be an imaging biomarker of the neuroinflammatory response ^5^ and neuroinflammation may lead to PHO development. PHO is thought evolve in stages with initial cytotoxic oedema leading to vasogenic oedema.

**The authors categorized inflammatory markers into six groups based on their biological properties: immune cells, cytokines/chemokines, tissue remodeling factors, damage-associated molecular patterns (DAMP), acute phase reactants, and adhesion molecules. However, the classification criteria may need reconsidering because some categories, such as acute phase reactants, may overlap. The authors should justify their classification system.**

 Response: Thank you for requesting this clarification. We have modified the text (Methods, eligibility criteria section): ‘We selected biomarkers if they were a marker (e.g., C-reactive protein) or a mediator (e.g., cytokines) of the inflammatory response and grouped the inflammatory biomarkers into six categories based on broad biological activity. Categories were defined based on discussions between domain experts (JB, BM). …immune cells, acute phase reactants (defined here as liver-produced plasma proteins (PMID: 21430962) cytokines/chemokines, damage-associated molecular patterns (DAMPs) (defined here as molecules released from intracellular compartments during inflammation (PMID: 25391648), tissue remodelling factors and adhesion molecules.

**The authors studied the connection between inflammatory markers and the prognosis of ICH, but they did not examine the relationship between PHO and ICH prognosis. The issue may be that various studies used different methods to measure PHO, so the differences in research approaches require clarification and summarization. The authors should investigate whether variations in research methods for measuring PHO could impact the prognosis of ICH.**

 Response: Thank you for this important comment. We did not examine how differences in the measurement of PHO between studies affected prognosis after ICH since this systematic review focused upon the relationship firstly, between serum biomarkers and PHO and secondly, between serum biomarkers and outcome rather than the relationship between PHO and outcome. However this has been discussed in other systematic reviews and meta analyses (Marchina et al. 2023: 10.1016/j.jstrokecerebrovasdis.2023.107204; Cliteur et al. 2023:  10.1177/23969873231157884) and we recognise this as a limitation: ‘We were unable to explore how variation in PHO measurement between studies affects any association between PHO and prognosis after ICH but this has been discussed elsewhere’ (Discussion)

**In their study, the authors found that MMP-9 showed a positive correlation with the volume of PHO and that MMP-2 and MMP-8 were positively correlated with the absolute volume of PHO. However, another study revealed that on the 7th day after ICH, MMP-2 had a negative correlation with PHO volume [3]. It is worth noting that the authors did not consider changes in the inflammatory response at different time points, which could affect the interpretation of the results. It is expected that there would be differences in each inflammatory marker at various stages of ICH.**  

 Response: Thank you for this comment. We now recognise this in the discussion: ‘There was heterogeneity in the timepoint of biomarker measurement which may also contribute to inconsistent findings since the level of a serum biomarker will vary in relation to the time of measurement after ICH onset.’

**According to the study, there is a connection between the volume of PHO and IL-10. However, previous research has stated that there is a negative relationship between PHO volume and IL-10 [4]. Another study found that higher levels of IL-10 in the blood are linked to a favorable prognosis 90 days after ICH. Additionally, administering IL-10 through the nose has decreased PHO and enhanced functional outcomes of mice with ICH [5]. As a result, the authors need to approach their findings with caution.**

 Response: Thank you for this important comment. We included reference 4, which the reviewer mentions above, in table 1, ‘Associations between circulating inflammatory biomarkers and perihaematomal oedema’ but incorrectly listed that it showed a positive correlation between IL-10 and PHO volume. This error may in part be due to the original paper incorrectly stating in the conclusion of the abstract that ‘serum levels of…..IL-10 are positively correlated.’ Thank you for pointing out that it actually demonstrated a negative relationship and we have corrected this both in table 1 and in the results section (where we have removed the reference to IL-10 in the sentence below): ‘IL-4 was positively associated with absolute PHO in one of two studies ^38^.

# Reviewer report: Reviewer response for version 1

**DOI:** 10.21956/wellcomeopenres.21268.r62058

**Published:** 2023-08-09

**Authors:** Felipe A. Montellano

**Affiliations:** 1 Department of Neurology, University Hospital Würzburg, Würzburg, Germany; 2 Institute of Clinical Epidemiology and Biometry, University of Würzburg, Würzburg, Germany

Thank you for the opportunity to comment on the manuscript by Kirby et al. on the association between blood-based inflammatory biomarkers and functional outcome and/or perihaematomal oedema after intracerebral haemorrhage. In their systematic review and meta-analysis, they summarize the results of 16 publications examining the association between 21 inflammatory biomarkers and perihaematomal oedema, and 93 studies examining the association between inflammatory biomarkers and clinical outcome. They conclude that higher circulating levels of WBC, CRP, fibrinogen and HMGB1 are associated with poorer outcomes after ICH. Authors are to be congratulated for the thoroughness of their work and for embarking on the always difficult task of summarizing the very heterogeneous evidence that abounds in the field of biomarkers and prognosis research. In my opinion, some points merit further discussion:

1. Risk of bias: Authors assessed risk of bias using a self-developed scale with elements from the Newcastle-Ottawa Assessment Scale, STROBE-ME and REMARK guidelines. While some relevant sources of bias are addressed by the proposed scale, others are not (particularly confounding, attrition, and statistical analysis). Further, as the authors rightly acknowledge in their limitations, a summed score probably does not fully reflect the risk of bias of certain studies. The QUIPS tool (Hayden JA et al. Ann Intern Med, 2013 ^1^) was specifically designed to assess the risk of bias in prognostic factor research. The authors may want to use this tool in their systematic review, at the very least for those articles undergoing meta-analysis. This might change the interpretation of some of the findings of the manuscript. As an example, most of studies reporting on the association between white blood cell count and functional outcome after ischaemic stroke (Montellano FA et al. Stroke, 2021 ^2^) arise from retrospective, convenience samples with suboptimal statistical modelling, lack of adjustment for relevant confounders and poor rates of follow up.
2. Meta-analysis: Following the recommendations of Riley et al. (BMJ, 2019) ^3^, it would be advisable to analyse hazard ratios, odds ratios, and risk ratios separately, as well as unadjusted and adjusted associations separately, instead of the reported standardised mean differences.

**Minor points**

1. Incremental value: Probably one of the most relevant questions in the field of prognosis research is whether novel prognostic factors, such as biomarkers, provide any incremental value on top of an established prognostic score , which often contains a set of readily available clinical variables (Steyerberg et al. PLoS Med. 2013 ^4^). It would be valuable if authors could report whether incremental value was investigated in the identified studies, either as improvement in discrimination, calibration, or overall model performance. Even if none of the identified studies addressed this issue, this would be a valuable insight. 
2. It would also be valuable if authors could report on the rate of studies adjusting for relevant confounding factors. At the very minimum, a valid association between a biomarker and poor outcome should be independent of age and clinical ICH severity.

Are the rationale for, and objectives of, the Systematic Review clearly stated?

Yes

Is the statistical analysis and its interpretation appropriate?

Partly

Are sufficient details of the methods and analysis provided to allow replication by others?

Yes

Are the conclusions drawn adequately supported by the results presented in the review?

Partly

Reviewer Expertise:

Stroke, prognosis research

I confirm that I have read this submission and believe that I have an appropriate level of expertise to confirm that it is of an acceptable scientific standard, however I have significant reservations, as outlined above.

## References

[1] : Assessing bias in studies of prognostic factors. *Ann Intern Med* .2013;158(4) : 10.7326/0003-4819-158-4-201302190-00009 280-6 10.7326/0003-4819-158-4-201302190-00009 23420236

[2] : Role of Blood-Based Biomarkers in Ischemic Stroke Prognosis: A Systematic Review. *Stroke* .2021;52(2) : 10.1161/STROKEAHA.120.029232 543-551 10.1161/STROKEAHA.120.029232 33430636

[3] : A guide to systematic review and meta-analysis of prognostic factor studies. *BMJ* .2019;364: 10.1136/bmj.k4597 k4597 10.1136/bmj.k4597 30700442

[4] : Prognosis Research Strategy (PROGRESS) 3: prognostic model research. *PLoS Med* .2013;10(2) : 10.1371/journal.pmed.1001381 e1001381 10.1371/journal.pmed.1001381 23393430 PMC3564751

# Response

**Published:** 2023-11-15

**Authors:** Caoimhe Kirby

**Affiliations:** DIIIRM, The University of Manchester, Manchester, England, UK

**Thank you for the opportunity to comment on the manuscript by Kirby et al. on the association between blood-based inflammatory biomarkers and functional outcome and/or perihaematomal oedema after intracerebral haemorrhage. In their systematic review and meta-analysis, they summarize the results of 16 publications examining the association between 21 inflammatory biomarkers and perihaematomal oedema, and 93 studies examining the association between inflammatory biomarkers and clinical outcome. They conclude that higher circulating levels of WBC, CRP, fibrinogen and HMGB1 are associated with poorer outcomes after ICH. Authors are to be congratulated for the thoroughness of their work and for embarking on the always difficult task of summarizing the very heterogeneous evidence that abounds in the field of biomarkers and prognosis research.**

 Thank you****

**In my opinion, some points merit further discussion:** **Risk of bias: Authors assessed risk of bias using a self-developed scale with elements from the Newcastle-Ottawa Assessment Scale, STROBE-ME and REMARK guidelines. While some relevant sources of bias are addressed by the proposed scale, others are not (particularly confounding, attrition, and statistical analysis). Further, as the authors rightly acknowledge in their limitations, a summed score probably does not fully reflect the risk of bias of certain studies. The QUIPS tool (Hayden JA et al. Ann Intern Med, 2013 1) was specifically designed to assess the risk of bias in prognostic factor research. The authors may want to use this tool in their systematic review, at the very least for those articles undergoing meta-analysis. This might change the interpretation of some of the findings of the manuscript. As an example, most of studies reporting on the association between white blood cell count and functional outcome after ischaemic stroke (Montellano FA et al. Stroke, 2021 2) arise from retrospective, convenience samples with suboptimal statistical modelling, lack of adjustment for relevant confounders and poor rates of follow up.**

 Response: Thank you for requesting this clarification.  We used a self-developed scale as although the STROBE-ME guidelines, Newcastle Ottawa scale and REMARK guidelines cover aspects of assessment of risk of bias we did not think that any of these three guidelines covered all the aspects that should be included (for example – the STROBE-ME and Newcastle Ottawa scale checklists do not include any aspects specific to biomarker measurement, while the REMARK checklist seems to place less emphasis on participant selection in comparison to the others but includes specific information relevant to serum biomarker studies.)  We have now also rated each of the studies included in a meta-analysis using the QUIPS tool and included this information in Supplemental Table 1 the extended data.  In accordance with Riley et al. (doi: 10.1136/bmj.k4597.) We selected a priori which domains we considered most important (study attrition, study participation and study confounding) and assigned each study an overall risk of bias based chiefly upon these domains. 12/20 (60%) had a moderate risk of bias, five studies (25%) had a low risk of bias and the remaining three (15%) had a high risk of bias. In the original submission if there were more  than  10  studies  included  in meta-analysis,   studies   we   stratified them into   high-quality   (0–1 risk of bias score) and low quality (≥2 risk of bias score) according to the eight item composite scale and performed sub-group analysis to   determine   the   impact   of study quality on summary estimates.  We have kept these analyses in the submission but have now also re-run our analyses of (1) the association between WBC and outcome, and (2) the association between CRP and outcome having stratified the studies by their risk of bias measured by the QUIPS tool (low/moderate/high) to determine the impact of study quality on the association between the biomarker and outcome. We have mentioned this in the methods section, page 5. 

**Analysis of WBC and outcome**:

 We have inserted a figure 2B into the supplement and inserted the following text into the results section, ‘immune cells’: Study quality did not influence pooled associations when risk of bias was assessed by the composite scale (p=0.07)  (Supplemental  Figure  2A ^13^) but when assessed by the QUIPS tool the association between WBC and outcome was no longer statistically significant in studies with a low risk of bias (pooled SMD 0.22; 95% CI -0.04-0.48).

**Analysis of CRP and outcome**:

 We have inserted a figure 5B into the supplement and inserted the following text into the results section, ‘acute phase reactants’: ‘Study quality when assessed both by the composite scale and by the QUIPS tool did not influence pooled associations (Supplemental Figure 5A and Figure 5B).’ We have also adjusted the abstract and the discussion accordingly to reflect the association between WBC and outcome no longer being significant in a meta-analysis of high quality studies.

**Meta-analysis: Following the recommendations of Riley et al. (BMJ, 2019) 3, it would be advisable to analyse hazard ratios, odds ratios, and risk ratios separately, as well as unadjusted and adjusted associations separately, instead of the reported standardised mean differences.**

 Response: Thank you for this comment. We selected studies for meta-analysis based upon those studies which had assessed the same biomarker and measured outcome in the same way at the same time point (Methods, meta-analysis section, page 3). Of those studies which contributed to any of the meta-analyses, none provided a hazard ratio. Only single studies reported odds ratios of the association between S100b and outcome, HMGB1 and outcome and fibrinogen and outcome. No studies of ferritin or TNFa provided an odds ratio. We have now included a sentence in the methods section: ‘We also used the stata command metan to perform meta-analysis of odds ratios where they were provided.’ Only one study provided an adjusted odds ratio (OR) but five studies provided an unadjusted odds ratio of the association between WBC and outcome and we have now included a meta-analysis of these studies (Results, immune cells section). ‘The pooled OR of five studies ^46,61 69,,81,86^ which reported an unadjusted odds ratio was 1.06 (95% CI 1.02-1.10)’ 4 studies provided an adjusted odds ratio of the association between CRP and outcome. 6 studies provided an unadjusted odds ratio of the association between CRP and outcome. We have added this information into the Results, acute phase reactants section. ‘The pooled adjusted OR of four studies, three of which had a low risk of bias according to QUIPS was 0.99 (95% CI 0.98-1.01). The pooled unadjusted OR of six studies, four of which had a low risk of bias according to QUIPS was 1.00 (95% CI 0.98-1.01).’ We have amended the discussion to reflect the result of the pooled analysis of ORs for the association between CRP and outcome. ‘CRP may be associated with poor outcome after ICH, ^127 ^but we did not find a consistent association.’ 2 studies provided an adjusted OR for the association between neutrophils and outcome and this information has been added (Results, immune cells section). ‘A pooled OR of two studies that provided an adjusted OR showed no association between neutrophil count and outcome  (OR 0.98, 95% CI 0.95-1.01).’ 1 study [Mrackova 2020] reported an unadjusted OR for the association between IL-6 and outcome and one study provided an adjusted OR for the association between IL-6 and outcome. This information has been added to the text (Results, cytokines/chemokines section).  

**Minor points** **Incremental value: Probably one of the most relevant questions in the field of prognosis research is whether novel prognostic factors, such as biomarkers, provide any incremental value on top of an established prognostic score , which often contains a set of readily available clinical variables (Steyerberg et al. PLoS Med. 2013 4). It would be valuable if authors could report whether incremental value was investigated in the identified studies, either as improvement in discrimination, calibration, or overall model performance. Even if none of the identified studies addressed this issue, this would be a valuable insight.**

 Response: Thank you for this important point.  We have added this into the Discussion, Limitations section. ‘None of the studies included in meta-analysis reported whether the biomarker helped to improve prediction of prognosis after ICH when compared or added to existing prognostic scores for ICH and this should be examined in future studies’.

**It would also be valuable if authors could report on the rate of studies adjusting for relevant confounding factors. At the very minimum, a valid association between a biomarker and poor outcome should be independent of age and clinical ICH severity.**

 Response: Thank you for this comment. We have included a sentence in each section commenting on this- WBCs: One of these studies (7.7%) included a multivariable analysis Neutrophil count: One study included a multivariable analysis CRP: Five of these studies (45%) included a multivariable analysis.  Fibrinogen: One study included a multivariable analysis Ferritin: Neither study included a multivariable analysis IL-6: Two studies (50%) included a multivariable analysis TNFa: No studies included a multivariable analysis S100B: Two studies (50%) included a multivariable analysis HMGB1: Neither study included a multivariable analysis
